# The Role of Cyclodextrins against Interface-Induced Denaturation in Pharmaceutical Formulations: A Molecular Dynamics Approach

**DOI:** 10.1021/acs.molpharmaceut.1c00135

**Published:** 2021-05-17

**Authors:** Marcello Rospiccio, Andrea Arsiccio, Gerhard Winter, Roberto Pisano

**Affiliations:** † Molecular Engineering Laboratory, Department of Applied Science and Technology, Politecnico di Torino, Corso Duca degli Abruzzi 24, Torino 10129, Italy; § Department of Pharmacy, Ludwig-Maximilians-University, 81377 Munich, Germany

**Keywords:** cyclodextrins, interface, protein stability, molecular dynamics, denaturation

## Abstract

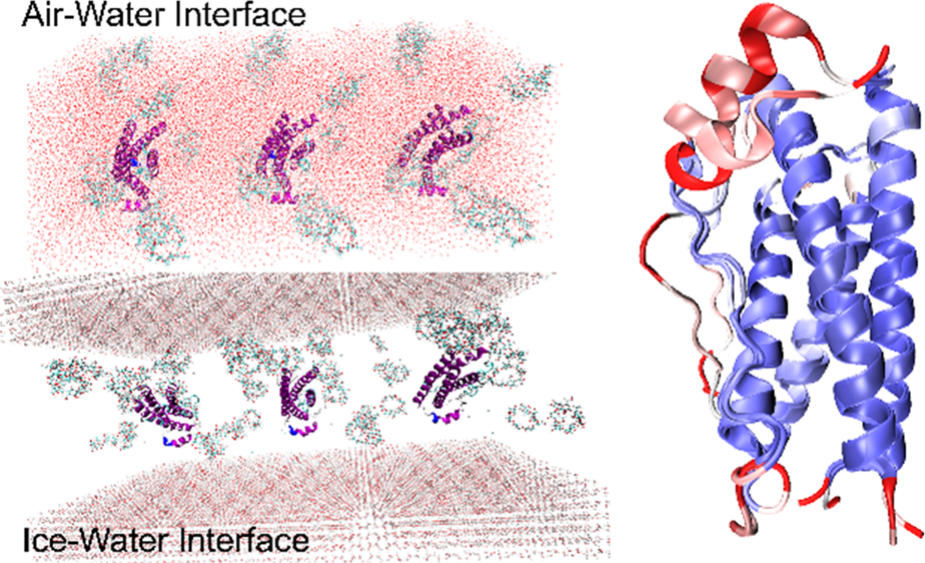

Protein-based pharmaceutical products are subject to a variety of environmental stressors, during both production and shelf-life. In order to preserve their structure, and, therefore, functionality, it is necessary to use excipients as stabilizing agents. Among the eligible stabilizers, cyclodextrins (CDs) have recently gained interest in the scientific community thanks to their properties. Here, a computational approach is proposed to clarify the role of β-cyclodextrin (βCD) and 2-hydroxypropyl-β-cyclodextrin (HPβCD) against granulocyte colony-stimulating (GCSF) factor denaturation at the air–water and ice–water interfaces, and also in bulk water at 300 or 260 K. Both traditional molecular dynamics (MD) simulations and enhanced sampling techniques (metadynamics, MetaD) are used to shed light on the underlying molecular mechanisms. Bulk simulations revealed that CDs were preferentially included within the surface hydration layer of GCSF, and even included some peptide residues in their hydrophobic cavity. HPβCD was able to stabilize the protein against surface-induced denaturation in proximity of the air–water interface, while βCD had a destabilizing effect. No remarkable conformational changes of GCSF, or noticeable effect of the CDs, were instead observed at the ice surface. GCSF seemed less stable at low temperature (260 K), which may be attributed to cold-denaturation effects. In this case, CDs did not significantly improve conformational stability. In general, the conformationally altered regions of GCSF seemed not to depend on the presence of excipients that only modulated the extent of destabilization with either a positive or a negative effect.

## Introduction

Therapeutic protein molecules are becoming increasingly important in the treatment of a large number of diseases, but they are often unstable and tend to undergo chemical or physical degradation.^1^ The 3D folded structure of proteins is easily affected by external stress, such as low/high temperature, extreme pH conditions, water removal, and exposure to interfaces.

The native conformation may be lost in these conditions, hence reducing the therapeutic potency. Partially unfolded or misfolded conformations may also enhance aggregation phenomena,^2,3^ and this poses serious safety issues, as the formation of aggregates may result in undesired immunogenicity.^4^

Among the possible sources of denaturation, the air–water and ice–water interfaces are commonly encountered during the production and storage of therapeutic proteins. The formation of a large air–water surface during mixing and shaking has often been shown to promote unfolding and aggregation.^5−9^ It is generally believed that the migration of proteins to the interface with air, as well as oil–water interfaces, where the exposure of the hydrophobic core is promoted, is responsible for the observed loss of stability.^10−13^ The formation of ice during freezing has also been found to be detrimental for proteins,^14−17^ but in this case, there still is no widespread agreement in the literature about the underlying mechanism. While it was first thought that adsorption onto the ice surface may be key for destabilization,^14,18^ recent experimental and simulation results indicate that direct interaction with the interface is not needed.^19−23^ In contrast, pressure build-up,^21^ concentration gradients and pH shifts,^21^ accumulation of gas bubbles,^24,25^ or cold denaturation phenomena^22^ were proposed as possible routes of denaturation upon ice formation. The addition of excipients to the protein formulation is therefore needed to prevent undesired loss of therapeutic potency and preserve the monomeric native conformation of the protein during both production and storage.

When surface-induced denaturation is an issue, non-ionic surfactants are often added to the formulation.^7−9,15,23^ These amphiphilic molecules are supposed to compete with the protein for interfaces, as such precluding protein adsorption.^26,27^ They also play a role in the aggregation pathway, probably by binding to the protein surface and hence preventing interactions.^7,28−30^

However, particle formation has recently emerged as a major concern in formulations containing some non-ionic surfactants, like the very common polysorbates.^31−34^ Polysorbates tend to degrade via autoxidation and hydrolysis, and this degradation leads to a buildup of various molecules that could potentially impact protein stability. For this reason, there is a demand for more stable excipients that can counteract protein aggregation, without posing problems of potential degradation of these excipients during long-term storage.

Among the possible candidates, the cyclodextrins (CDs) represent an interesting class of molecules.^35,36^ They are cyclic oligosaccharides composed of α-glucopyranose monomers and can contain six (αCD), seven (βCD), or eight (γCD) monomeric units. CDs are characterized by a unique torus-like shaped structure, with a hydrophilic outer surface and an internal hydrophobic cavity, surrounded by two rims (primary rim, formed by C6 atoms, and secondary rim, consisting of the C2 and C3 glucose atoms). βCD, which comprises seven glucose units, gained a lot of attention because of its hydrophobic cavity diameter, which allows a good fit of aromatic amino acids. The inclusion of some residues, such as Phe, Tyr, His, and Trp, within the cavity and the consequent formation of protein–CD complexes are supposed to efficiently prevent aggregation.^37−40^ The low aqueous solubility of βCD (16 mM at 25 °C) makes it unsuitable in parenteral formulations, but the substitution of some hydroxyl groups with other moieties can ameliorate this issue. For instance, an important group of βCD derivatives involves hydroxypropyl groups linked to the glucose monomers.^41^ The resulting hydroxypropyl-βCD (HPβCD) displays greater solubility and is already used in the formulation of approved parenteral products. Moreover, HPβCD has also been reported to be surface-active, competing with the protein for the air–water interface and preventing agitation-induced aggregation.^42,43^ However, it was also found that HPβCD could not displace proteins from the interface as efficiently as classical surfactants do,^44−46^ meaning that its stabilizing effect should be mostly attributed to protein–cyclodextrin interactions rather than to its weak surface activity.

**Figure 1:**
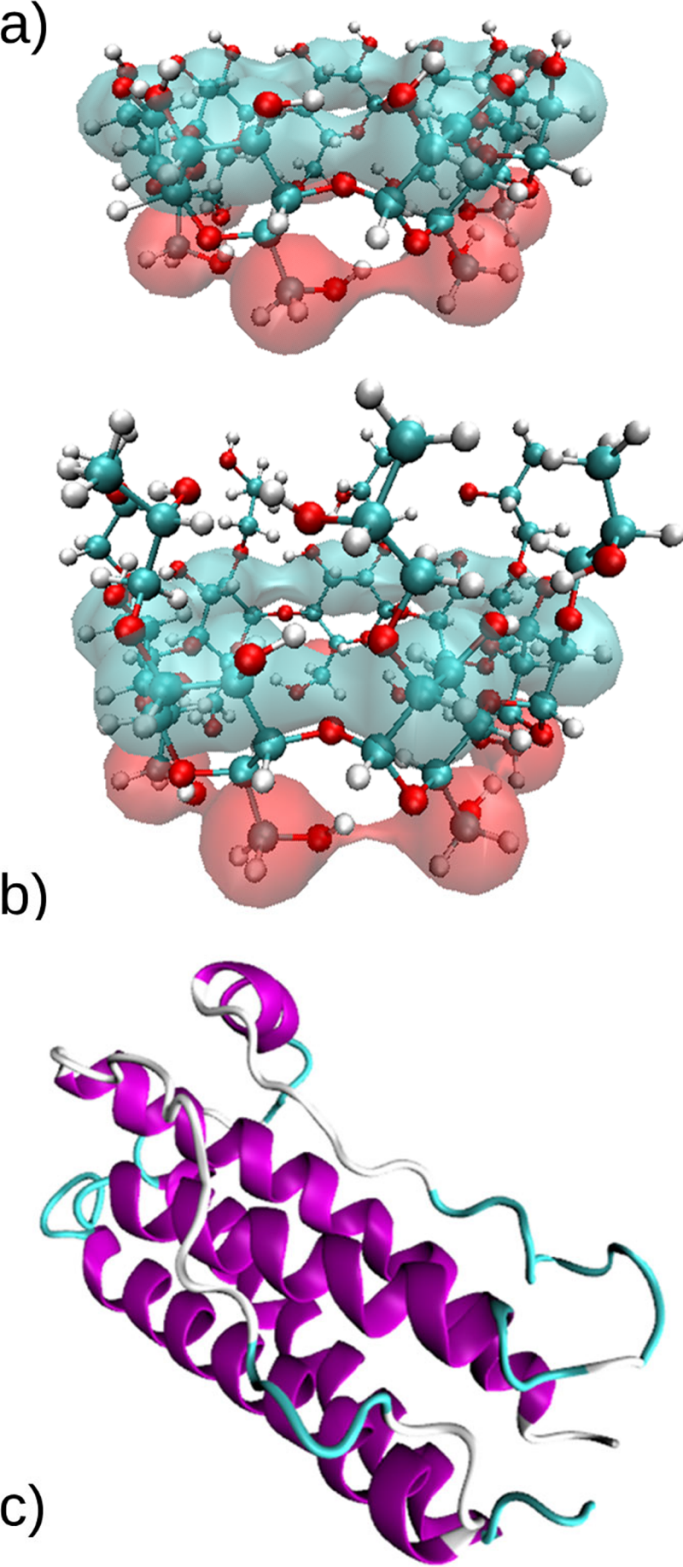
Snapshots of (a) β-cyclodextrin and (b) 2-hydroxypropyl-β-cyclodextrin. The red surface encompasses the primary rim, while the light-blue one delimitates the secondary rim. (c) Granulocyte-colony stimulating factor. The different colors identify different secondary structures. Purple: α-helix, cyan: turn, white: coil.

We will here focus our attention on HPβCD and compare it with the non-substituted βCD. Different possible forms of HPβCD exist, depending on the degree of substitution and position of the derivatization, and we here selected the form where the hydroxypropyl group is linked to the O_2_ atom of the glucose unit (2-HPβCD) and fully substituted for all seven residues. A snapshot of the CD molecules investigated in this work is shown in Figure 1a,b, where the primary and secondary rims have been highlighted.

The objective of this work is to clarify the molecular mechanism at the basis of CD-induced stabilization of proteins at the air– and ice–water interfaces. For this purpose, a molecular dynamics (MD) investigation will be performed. MD is a powerful tool for the analyses of molecular interactions, and will here be used in its all-atom variant, with an explicit treatment of water molecules. Enhanced sampling techniques, such as the well-known metadynamics (MetaD) approach,^47^ will also be used to enhance the exploration of the free energy landscape and overcome the timescale limitations of classical MD simulations.

Granulocyte colony-stimulating factor (GCSF) will be used as model protein for this investigation, because its behavior is well-known experimentally.^48,49^ A cartoon representation of this molecule is shown in Figure 1c. Overall, our results will confirm the amphiphilic properties of HPβCD, and prove its superior properties compared to the unsubstituted βCD. Preferential orientation of the rims to the air-water and ice-water interfaces will be discussed. In line with previous observations, we will demonstrate that no significant protein or CD adsorption occurs at the interface with ice.

## Methods

### Simulation Details

All MD simulations were performed using GROMACS 2018.6.^50^ The CHARMM36m force field was used for the protein^51^ in combination with explicit CHARMM TIP3P water.^52^ The CHARMM36 force field was used to describe the β-cyclodextrin,^53^ while the hydroxypropyl derivatization was modeled with parameters obtained from SwissParam.^54^ Periodic boundary conditions were used for all systems. Long-range electrostatics interactions were evaluated with the PME approach.^55^ A cut-off radius of 1.2 nm was used for both Coulomb and Lennard–Jones potentials.

The configuration file 1CD9^56^ for the GCSF was obtained from the RCSB PDB data bank.^57^ The protein was simulated starting from the native configuration, both in the presence and absence of excipients. The protonation state of the different residues was adjusted to a value corresponding to pH 4.5, using the H++ server, version 3.2 (http://biophysics.cs.vt.edu/H++).^58^ To ensure neutrality of the system, Cl^–^ ions were added to the solution, balancing the charge carried by GCSF (+ 7).

The GenIce algorithm^59^ was used to obtain the configuration of hexagonal (Ih) ice, with an 8.6 × 8.1 × 2.7 nm^3^ (7.8 × 8.1 × 2.7 nm^3^ for simulation (sim.) 2) size, which was then oriented with the basal {0001} plane toward the liquid phase. The Ih ice water molecules were kept frozen in place during the simulations.

Conditions and details about the systems studied in this work are summarized in Table 1. All systems were energy-minimized using the steepest descent algorithm and subsequently equilibrated in the NPT ensemble with the Berendsen thermostat–barostat^60^ coupling for 1 ns. For simulations 1, 7, 8, 15, and 16 in Table 1, the first equilibration did not involve the presence of the air interface yet, and the bulk solution only was brought to the desired values of temperature and pressure. Afterward, a 4.8 nm (2.1 nm for simulation 1) vacuum space was added along the *z* axis above the liquid phase, and the production run was subsequently performed in the NVT ensemble, controlling temperature with the V-rescale thermostat.^61^ All the other simulations were performed in the NPT ensemble controlling pressure with the Parrinello–Rahman barostat.^62^ For systems involving an ice layer (2, 9, 10, 17, 18), the barostat used during both equilibration and production was semi-isotropic, so as to hold the *xy* box dimensions fixed, while the *z* dimension was allowed to fluctuate. For controlling temperature in simulations 3, 4, 5, and 6 the Nosé–Hoover thermostat^63−65^ was used, while the V-rescale thermostat^61^ was employed for all the other systems. The production run was performed for the duration listed in Table 1, and the time-step used in all simulations was equal to 2 fs.

**Table 1:**
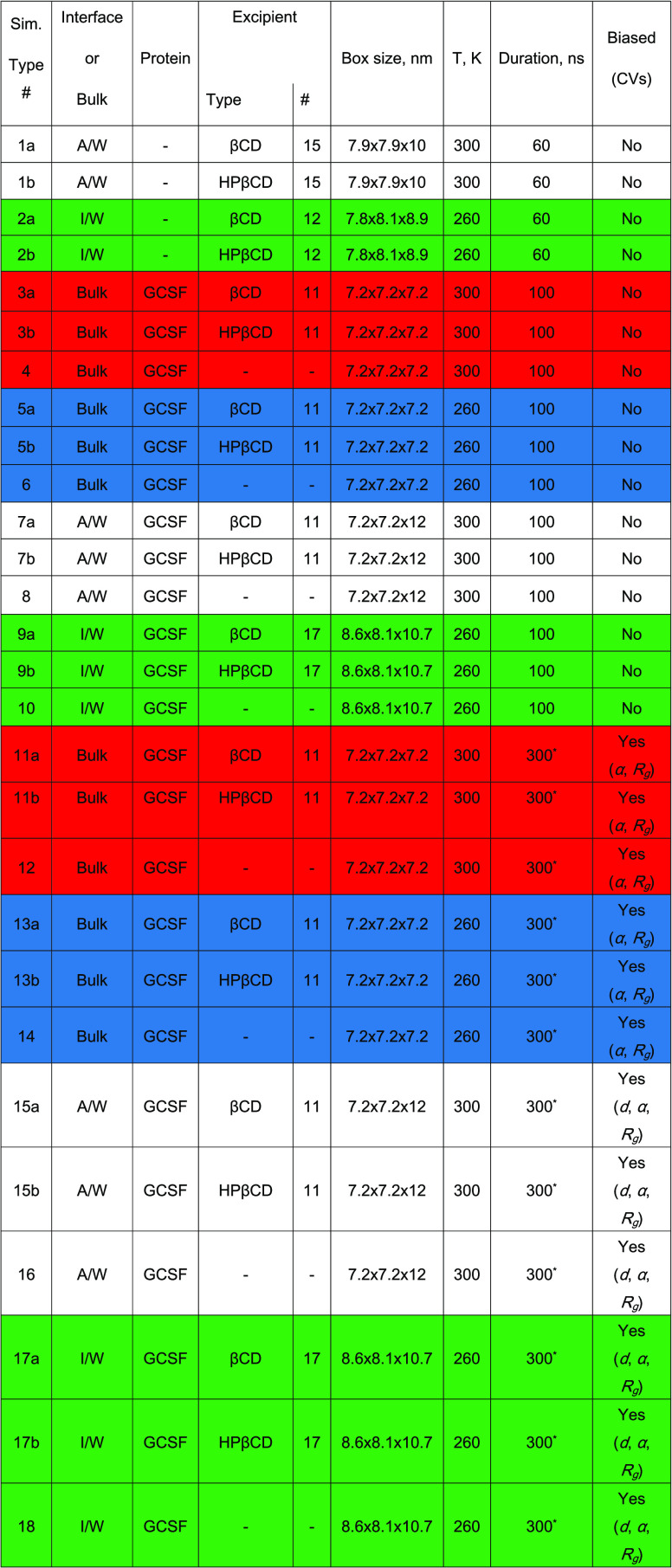
Summary of the Simulations Details^a^

a A/W: air–water, I/W: ice–water. Color code: white, A/W interface; red, aqueous bulk, 300 K; blue, aqueous bulk, 260 K; green, I/W interface. *, Biased simulations (11–18) have an overall duration of 300 ns, 100 ns for each walker.

The number of CDs in each system was varied (see Table 1) in order to keep a constant concentration of 50 mM, which is already used in some commercial pharmaceutical products.^35^ This concentration was used for βCD as well, despite being higher than the solubility limit at room temperature. No precipitation was anyway observed during the simulated time, and the choice to work beyond the solubility value was made to guarantee a statistically relevant number of CD molecules in each box. Working below the solubility limit, with a too small number of excipient molecules in each box, would have made the computation of simulated properties statistically unreliable. The trajectories were visualized using Visual Molecular Dynamics (VMD),^66^ version 1.9.3.

To verify the convergence of unbiased simulations (sims. 1–10), we evaluated the time evolution of the number of CDs within 2 nm from the GCSF surface, or from the ice surface. These results can be found in Figure S1 of the Supporting Information.

### Parallel Bias Metadynamics

In order to overcome the limitations of traditional MD simulations, enhanced sampling techniques were used^67^ in sims. 11–18. Specifically, among the possible alternatives, parallel bias metadynamics^68^ (PBMetaD) was chosen. PBMetaD speeds up the sampling by simultaneously applying different mono-dimensional bias potentials, acting on selected degrees of freedom of the systems, generally referred to as collective variables (CVs). Also, three multiple walkers^69^ (MWs) were used for each simulation. Each walker was simulated for 100 ns, so as to obtain a 300 ns total sampling time.

Simulations were performed with PLUMED 2.5.1,^70,71^ patched to GROMACS 2018.6. The chosen CVs were the radius^72^ of gyration (*R*_g_), the α-helix content^73^ (α), and the distance (*d*) of the center of mass of the protein from the interface. The initial configuration of each system was the last frame of the corresponding unbiased simulation (sims. 3–10 in Table 1). The bias factor was equal to 15; the initial Gaussian height was set to 2 kJ/mol; and the Gaussian deposition rate to 1 hill/ps. Further details about the simulations are listed in Table 1. Other parameters for these simulations are the same already described in the Simulation Details section. Finally, the free energy surfaces (FES) and the probability distributions of the CVs were obtained by using the reweighting technique proposed by Tiwary and Parrinello.^74^

To verify the convergence of the biased simulations (sims. 11–18), we computed the variation of the CVs over the last 10% of the simulation time. These results are reported in Figure S2 of the Supporting Information.

### Analyses of the Trajectories

#### Distance Root Mean Square Deviation (dRMSD)

The distance root mean square deviation (dRMSD) was calculated with respect to the backbone of the native structure of GCSF (1CD9^56^ configuration file from the RCSB PDB^57^ data bank). The expression for the calculation of dRMSD implemented by PLUMED is the following:^71^
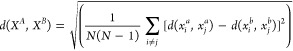
1where *X^A^* and *X^B^* are the structures to be compared, *N* is the number of atoms, and *d*(*x_i_* , *x_j_*) represents the distance between atoms *i* and *j* within the same structure. To reduce the computational cost of these calculations, both an upper and a lower cut-off were used, which are 0.1 and 3.0 nm, respectively. This means that only pairs of atoms whose distance, in the reference structure, was within such limits were considered.

#### Cluster Analysis

The protein conformations during the biased trajectories (11–18) were grouped together by performing a cluster analysis based on the Daura algorithm.^75^ The conformations were grouped together if the root mean square deviations of the N–C_α_–C atoms were less than 0.1 nm compared to each other. For this analysis, the trajectories were previously reweighted according to the technique by Tiwary and Parrinello.^74^

## Results and Discussion

### HPβCD Shows Stabilizing Properties at the Air–Water Interface but Is Not Attracted by Ice

The preferential interaction/exclusion behavior of CDs toward GCSF was first evaluated. CDs were generally found to be preferentially included within the protein hydration layer (see the Supporting Information section S2, and Figures S3 and S4). The inclusion of protein residues within the CD cavity was also addressed, and solvent accessibility was found to be key for the formation of inclusions. Moreover, CDs included not only aromatic sidechains (a well-known observation in the literature), but also other types of residues, suggesting a favorable interaction of the backbone group with the hydrophobic cavity of CDs. More details on this analysis can be found in the Supporting Information file section S3 and Figures S5–S8.

Here, we will now focus our attention on the behavior of CDs and GCSF in the presence of interfaces, starting from the air–water surface (sims. 1, 7, and 8). The normalized density profiles of CDs alone, without protein (sim. 1, Figure S9a,b), showed accumulation of HPβCD at the interface, with a preferential orientation of the secondary rim (Figure S9b), thus of the hydrophobic cavity, toward the gaseous phase. Differently, the βCD molecules accumulated in bulk, with no preferential orientation (Figure S9a). Snapshots of the systems are shown in Figure S9c,d.

**Figure 2:**
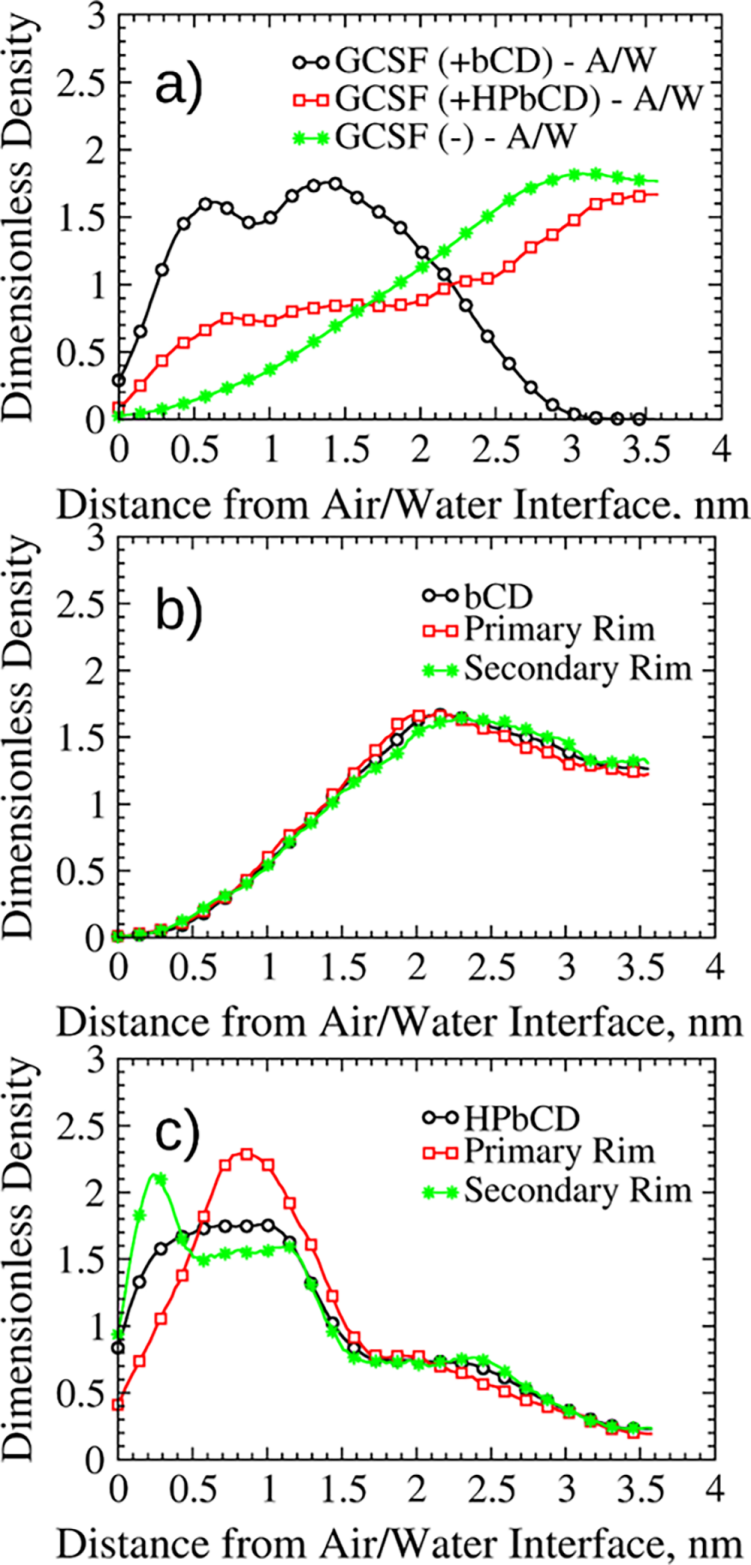
Normalized density profiles for the GCSF formulations at the air–water interface. (a) Protein profiles (sims. 7, 8). (b) Density profiles for βCD and its rims (sim. 7). (c) Density profiles for HPβCD and its rims (sim. 7).

The behavior of GCSF at the air–water interface was also evaluated, both with and without excipients (sims. 7, 8). The density profiles of the protein (Figure 2a) mainly showed accumulation in the aqueous bulk. Nonetheless, in all three cases, the density of the protein at the interface was not equal to zero, meaning that there were interactions between GCSF and the surface. However, in the presence of βCD the accumulation in proximity of air was more pronounced, suggesting that the native CD, repelled by the surface, promoted the protein–surface interaction. The exclusion of βCD from the air–water interface, and its consequent fostering of protein–air interaction, represents a radical difference of this molecule compared to surfactants.

The density profiles of the CDs are not dramatically perturbed by the addition of the protein, and adsorption at the interface with air was again observed for HPβCD (as evident comparing Figure S9b and Figure 2c), with preferential orientation of the secondary rim toward the surface (Figure 2c). In contrast, βCD accumulated in bulk (Figure 2b). Snapshots of these systems are illustrated in Figure S10.

These preliminary simulations confirm the stabilizing properties of HPβCD at the air–water interface, as observed in the literature.^43^ Also, the addition of HPβCD resulted in a slightly reduced radius of gyration of GCSF at the air–water interface (this observation will be further discussed in the following). The radius of gyration is a measure of protein size, and an increase in its value corresponds to a loss of compactness, which is often associated to the unfolding process. These results can be rationalized assuming that HPβCD protects GCSF from the loss of structure that may occur at the interface with air by reducing protein adsorption.

Overall, βCD showed destabilizing properties, pushing GCSF closer to the air–water interface and causing adsorption. The native CD, displaced from the surface and forced to accumulate within the bulk solution, formed an increased number of clusters (the 11 CD molecules clustered into aggregates containing, on average, 4.13 molecules each), and this contributed to its decreased interaction with the protein. Conversely, HPβCD showed stabilizing properties by both adsorbing at the air–water interface and forming inclusions with non-polar residues (see the Supporting Information file, section S3).

**Figure 3:**
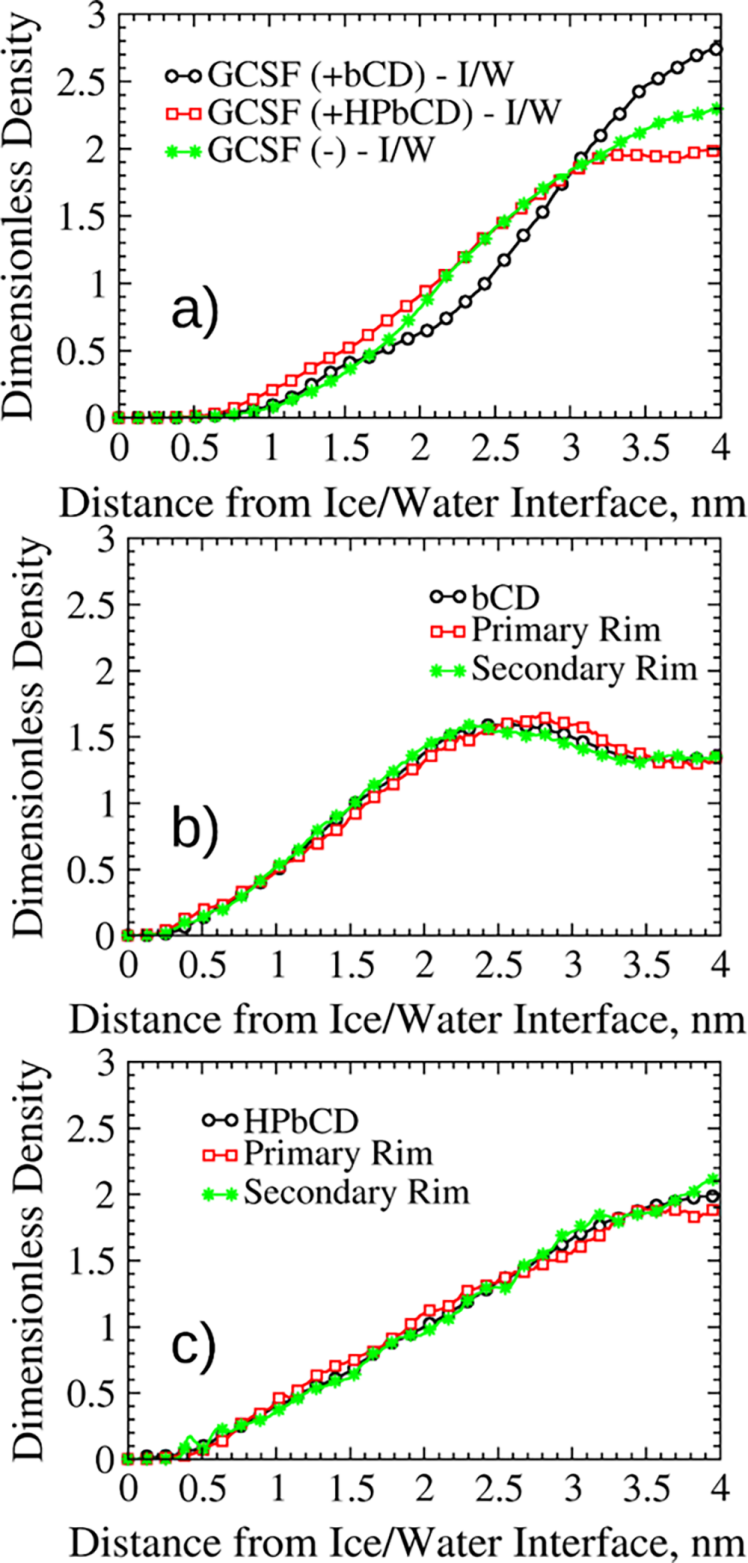
Normalized density profiles for the GCSF formulations at the ice–water interface. (a) Protein profiles (sims. 9, 10). (b) Density profiles for βCD and its rims (sim. 9). (c) Density profiles for HPβCD and its rims (sim. 9).

The situation changes considerably at the ice–water interface (sims. 2 and 9). The density profiles of the ice–water systems without GCSF (sim. 2) showed that both CDs accumulated in bulk (Figure S11a,b), with no preferential orientation of their rims. The same behavior was observed for the CDs in the systems with the protein (sim. 9, Figure 3b,c). GCSF accumulated in the bulk, as well (Figure 3a).

However, since GCSF did not exhibit prominent adsorption to ice, the apparent lack of surfactant-like properties showed by the CDs in presence of the ice interface should not invalidate their potential as excipients.

**Figure 4:**
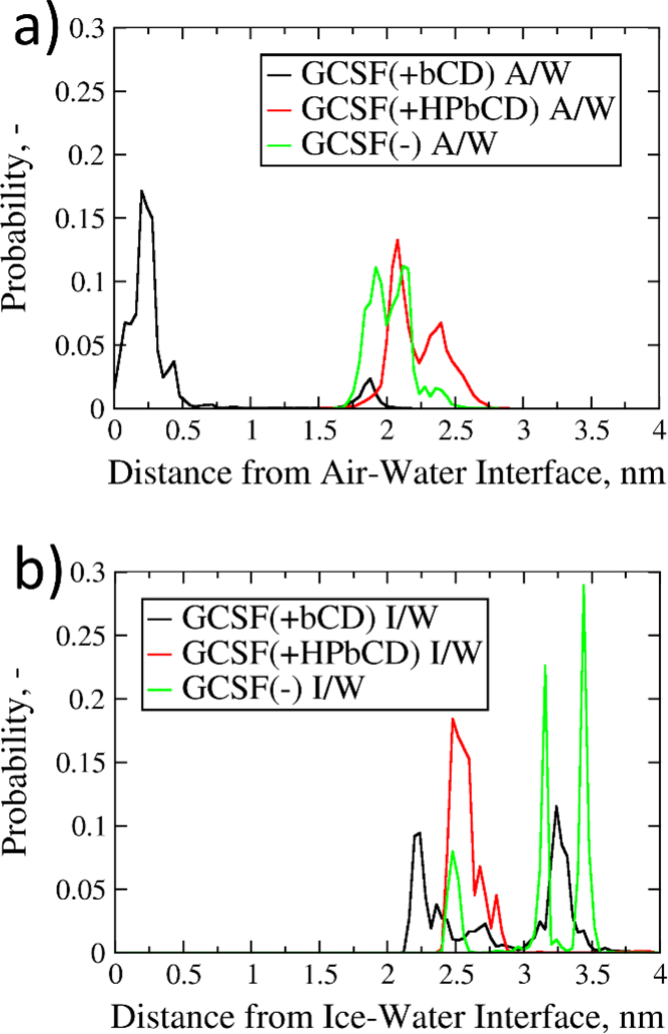
Probability distributions of the interface-GCSF distance. (a) Air–water systems (sims. 15 and 16 in Table 1). (b) Ice–water systems (sims. 17 and 18 in Table 1).

Some more insight into the stabilizing action of the CDs can be obtained from our PBMetaD simulations (15–16 from Table 1), where the improved sampling allows the observation of unfolding transitions. GCSF tended to be confined close to the air–water interface in the presence of the native CD, while in the other two cases (i.e., absence of excipients or addition of HPβCD), the protein spent most of the time in bulk (Figure 4a). The ability of HPβCD to stabilize GCSF at larger distances from the air surface was evident. This behavior may be explained either by the preferential adsorption of HPßCD at the interface, preventing or obstructing air–protein interactions, and/or by direct interaction with the peptide molecule^44−46^ (see the Supporting Information, sections S2 and S3). The range of α-helix content sampled during simulations 15 and 16 was quite similar for all systems (Figure S12), although it seems that protein conformations with lower helicity were most likely observed in the presence of βCD (Figure S12a) and in proximity of the air surface. For the other two systems, some secondary structure loss was also observed but was not related to the presence of the interface (Figure S12b,c).

At the ice–water interface, GCSF did not approach the ice surface in any of the systems investigated (Figure 4b), confirming the predictions of unbiased MD simulations (Figure 3a). At a larger distance from the surface, some conformations with lower α-helix content were sampled in the presence of HPβCD (Figure S13c) and, in a more limited way, also without excipients (Figure S13b). In the presence of the native CD, the α-helix content was less likely to undergo transitions (Figure S13a), as energy barriers seemed to be sharper, if compared to the other two systems.

### Conformational Behavior of GCSF

In order to investigate the conformational behavior of GCSF, further analyses were performed. More specifically, the evolution of the radius of gyration and dRMSD of GCSF was extracted from the simulations, analyzed, and compared for different systems.

**Figure 5:**
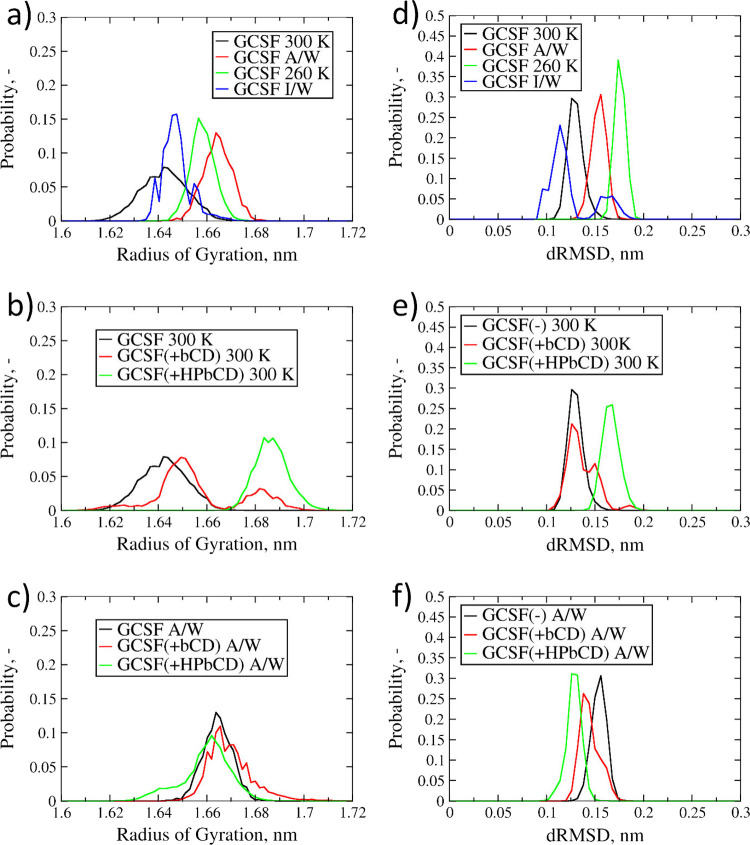
Probability distributions of *R*_g_ and dRMSD values for GCSF. (a, d) GCSF systems without excipients (sims. 12, 14, 16, and 18 in Table 1). (b, e) Systems in aqueous bulk at 300 K (sims. 11 and 12 in Table 1). (c, f) Systems at the air-water interface (sims. 15 and 16 in Table 1).

We will first focus our attention on the systems without CDs (Figure 5a,d) in order to understand the effects of both temperature and interfaces. The comparison between the two protein systems (300 and 260 K) in aqueous bulk allowed us to highlight the effect of temperature on the conformational stability. In fact, both the *R*_g_ (Figure 5a) and dRMSD (Figure 5d) distributions at 260 K were shifted to higher values compared to ambient temperature (300 K), i.e., larger conformations could be more easily sampled at low temperature. This result may seem counterintuitive, as conformational sampling and water diffusivity are reduced at low temperature. However, energy barriers were lower at 260 K, promoting conformational changes. This slight structure expansion at 260 K may suggest the onset of cold denaturation effects.

For what concerns the air–water interface, the *R*_g_ distributions (Figure 5a) confirmed the detrimental effect of this interface on the protein conformational stability.

Finally, in presence of the ice–water interface, the distribution of radius of gyration values was shifted toward slightly lower values compared to those at 260 K in the aqueous bulk (Figure 5a). However, the *R*_g_ values sampled in this condition were still slightly higher than those at ambient temperature (300 K).

We will now focus our attention on the effects of CDs on the conformational stability of GCSF. In the aqueous bulk at 300 K, the dRMSD distribution for the βCD system was analogous to the case without excipient (Figure 5e), while the radius of gyration presented slightly higher values (Figure 5b). Conversely, both the dRMSD and *R*_g_ values for the HPβCD systems were distinctly higher. It would hence seem that HPβCD caused a reduction of conformational stability, as it was observed experimentally for IgG formulations.^46^ This reduction in stability may be correlated to the inclusions within the CD’s hydrophobic cavity discussed in the Supporting Information, section S3. Specifically, we found that the residues most likely to be included into the CDs cavity were not necessarily the hydrophobic ones. The hydrophobic residues have high affinity for CDs cavity but are normally buried in the protein core, and as such, their inclusion is statistically unlikely. Only when unfolding occurs such hydrophobic residues become surface exposed and can be included within CDs cavities. The exposure of hydrophobic residues, and subsequent inclusion within the hydrophobic cavity, may be the driving force for the conformational changes induced by HPβCD. However, such changes were quite modest in absolute terms. The behavior observed in the presence of HPβCD could be explained by the smoother energy profile towards higher values of *R*_g_, while for the other two systems, an increase in radius of gyration was energetically hindered.

At the air–water interface, both the dRMSD and *R*_g_ (Figure 5c,f) distributions of the βCD system were roughly in the same range of the protein system; however, the values were higher than those of the corresponding bulk case (Figure 5b). Such results were somewhat expected, as they are fully in agreement with the interface-distance distributions (Figure 4a), further confirming the scarce ability of βCD to counteract interface-driven denaturation in the presence of air. In fact, the native CD seemed less likely to preserve structural integrity and compactness at the interface because it promoted protein adsorption (as discussed previously) and, consequently, increased the risk of surface-induced denaturation.^76,77^ On the contrary, the dRMSD distribution in the presence of HPβCD was shifted to considerably lower values (Figure 5f), while the *R*_g_ profile was less affected (Figure 5c). HPβCD showed a clear stabilizing action toward the protein, increasing the conformational stability and preventing its adsorption to the surface (Figure 4a).

**Figure 6:**
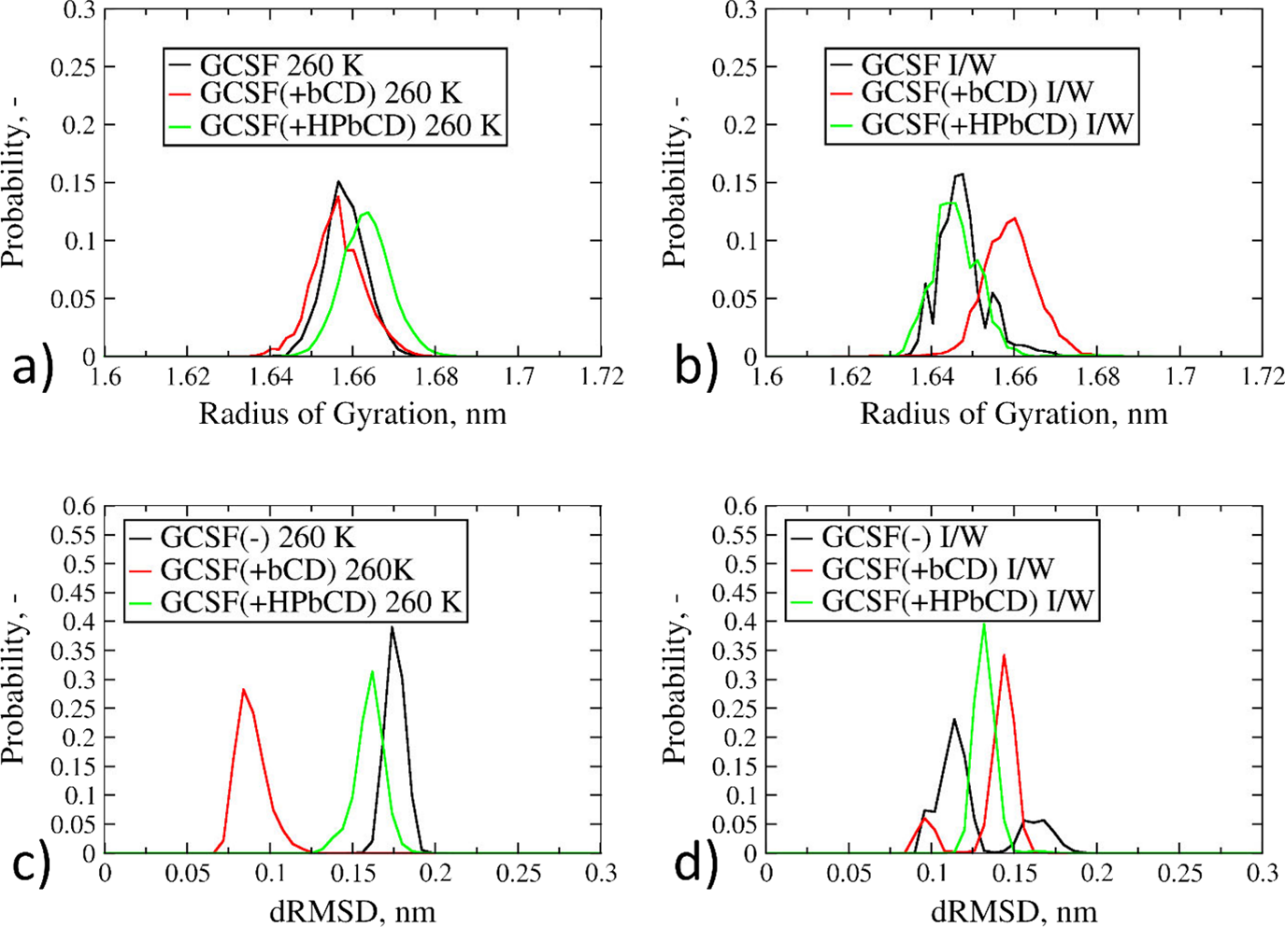
Probability distributions of *R*_g_ and dRMSD values for GCSF. (a, c) Systems in the aqueous bulk at 260 K (sims. 13 and 14 in Table 1). (b, d) Systems at the ice-water interface (sims. 17 and 18 in Table 1).

We have already discussed the destabilizing effect of lower temperatures for the GCSF system in aqueous bulk at 260 K. The addition of HPβCD did not seem to introduce any advantage in terms of stabilization (Figure 6a,c). Instead, βCD was able to reduce the dRMSD values (Figure 6c); however, no improvements in terms of radius of gyration were observed (Figure 6a).

Finally, the effect of the ice–water interface on the conformational stability of GCSF presented some mild differences with respect to the previously discussed case. For both CDs, the most likely dRMSD values were slightly higher than those of the excipient-free system (Figure 6d). Instead, when considering the radius of gyration, the behavior of the two excipients was different: the addition of βCD favored more expanded protein structures, while HPβCD did not show any meaningful change with respect to the stand-alone GCSF (Figure 6b). Overall, the native CD had a destabilizing effect on both compactness and conformational stability at the ice interface, while GCSF, both by itself and in the presence of HPβCD, seemed to be rather stable.

### GCSF Conformational Transitions Involve a Few Protein Regions, and Cyclodextrins Modulate the Extent of These Structural Changes

After having analyzed the conformational stability of the protein as a whole, we focused our attention on the role of specific peptide sequences. Proteins are characterized by complex structures, and specific regions or sequences of amino acids may be crucial for what concerns denaturation and/or aggregation. Therefore, we decided to assess the contribution of different residues to the global behavior of GCSF. Specifically, we tried to determine if CDs were able to modify the behavior of specific patches on the surface of GCSF,^78^ for example, because of mutual interactions.

We used the algorithm proposed by Daura et al^75^ to identify the most sampled protein conformations for each PBMetaD simulation (simulations 11–18 from Table 1).

The most probable structures for each system were aligned to the most sampled conformation of GCSF at 300 K in aqueous bulk, using the STAMP algorithm^79^ as implemented in the MultiSeq^80,81^ tool of VMD.^66^ The aligned structures were then colored according to the *Q*_res_ value,^81^
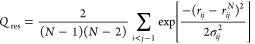
2where *N* is the number of residues of the protein, *r_ij_* is the distance between a pair of *C*_α_ atoms, *r^N^_ij_* is the *C_α_* – *C_α_* distance between residues *i* and *j* of the aligned structures, and σ*^2^_ij_* (that is equal to |*i – j*|^0.15^) is the standard deviation, which determines the width of a Gaussian function.^81^

*Q*_res_ represents the degree of conformational similarity of the aligned structures. Its value ranges between 0 and 1, where 1 (purple color) indicates complete structural similarity, while 0 (red color) stands for absence of similarity. The aligned structures, colored according to the *Q*_res_, are shown in Figure S14.

**Figure 7:**
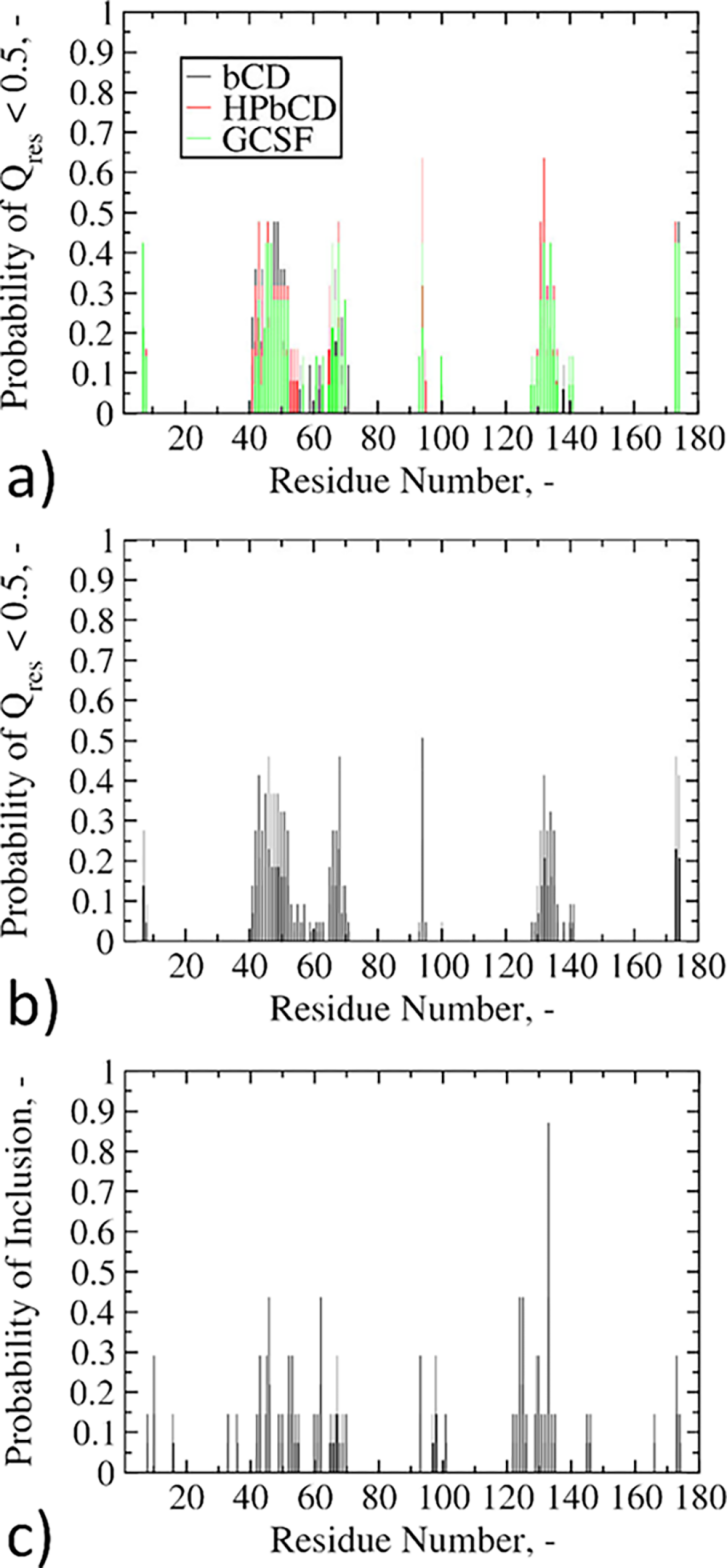
Probability distributions for GCSF residues (ranging from 7 to 174). (a) Residues with a *Q*_res_ lower than 0.5, distinguishing the type of excipient (sims. 11–18 in Table 1). (b) Residues with a *Q*_res_ lower than 0.5, averaging all the systems together (sims. 11–18 in Table 1). (c) Residues included by CD hydrophobic cavities (simulations 3, 5, 7, and 9 in Table 1).

We then identified the residues that were mostly involved in conformational changes, based on the value of *Q*_res_. Specifically, residues with a *Q*_res_ lower than 0.5 were considered to represent the most unstable patches of GCSF. This analysis was performed separately for systems containing βCD, HPβCD, or without excipients. The results are shown in Figure 7a.

This figure shows that the three distributions (excipient-free, βCD, or HPβCD) were substantially overlapping. This result indicates that the amino acids having the largest influence on the conformational stability of the protein (SER7, CYS42-HIS52, SER66-ALA68, GLY94, GLN131-GLY135, GLN173-PRO174) are fundamentally the same in every system under consideration. In other words, CDs modulate only the extent of the conformational changes, without determining the regions of the protein involved.

As a further step, we collapsed the three distributions of Figure 7a into one distribution only, which is averaged over all systems (Figure 7b). This latter distribution was subsequently cross-referenced with the protein patches that are most frequently included within the CD hydrophobic cavities (Figure 7c, further details on the evaluation of inclusions can be found in the Supporting Information, section S3). Figure 7b,c is once again almost superimposable, with only minor differences in terms of frequencies. These results seem to suggest that inclusions do not actually exert significant influence over the conformational stability of GCSF, as the same residues included within the CD cavity were involved in conformational changes even in the absence of excipients.

Finally, the *Q*_res_ distribution was compared to the aggregation prone regions (APRs), as obtained using the AMYLPRED2 server.^82^ The protein sequences PHE13-CYS17, CYS36-LYS40, LEU47-SER53, ILE56, SER80-LEU89, PHE113-TRP118, PHE140-ALA143, and VAL151-HIS170 were identified as APRs. Although some of the residues showed correspondence, in most cases, these amino acid sequences did not match with low values of *Q*_res_, therefore suggesting that the APRs do not necessarily coincide with conformationally destabilized regions.

The last objective of these analyses was to investigate the nature of the residues (in terms of polarity/charge and amino acid type) that mostly affected the conformational stability of GCSF. We found that the most recurrent amino acids are also some of the most abundant in terms of GCSF composition. Specifically, all classes of amino acids contributed to conformational changes, and the most frequent were LEU, GLN, GLU, PRO, GLY, SER, HIS, and ALA.

## Conclusions

We used a computational approach to investigate the properties of two eligible excipients, βCD and its derivative HPβCD, against the surface-induced denaturation of GCSF. Initially, MD simulations were performed to assess the equilibrium distribution of cyclodextrins at the air–water and ice–water interfaces. HPβCD was found to accumulate at the air–water interface, with its cavity oriented toward the gaseous phase. The interactions between GCSF and CDs were also investigated. The CDs were observed to be preferentially included in all systems studied, with βCD generally being more included than HPβCD. Inclusion of peptide residues was observed in all cases not only for aromatic species but for all kinds of amino acids. This suggests a mechanism based on the interaction between the backbone of the protein and the cyclodextrin hydrophobic cavity.^35^

The native CD promoted protein adsorption at the air–water interface, while the functionalized one was able to accumulate at the interface, favoring the accumulation of GCSF in the bulk of the solution. The interaction of HPβCD with GCSF molecules that are present at the surface and that underwent some conformational changes may be responsible for the mitigation of aggregation phenomena observed in experiments. No interaction between the protein and the ice–water interface was observed, nor between CDs and ice.

PBMetaD simulations were also performed to verify the validity of these observations to longer timescales. The enhanced sampling trajectories confirmed that HPβCD was able to prevent protein adsorption at the air–water interface, while βCD displayed destabilizing properties.

Conformational changes in the bulk systems were enhanced at low temperature (260 K), which may be indicative of cold denaturation. In contrast, the ice–water interface seemed not to exert a dramatic effect on GCSF.

At 300 K and in the absence of interfaces, CDs did not improve conformational stability, and HPβCD even had a deleterious effect (as already observed experimentally in IgG formulations^46^).

Finally, we observed that the sequences of peptide residues mostly involved in conformational transitions of GCSF were the same for all systems, independently of the presence of excipients. In this sense, CDs seemed to modulate only the extent of conformational changes. The residues involved in conformational changes coincided with the most abundant ones in terms of GCSF amino acid composition, and generally did not coincide with aggregation prone regions.

Overall, HPβCD seems a viable excipient and the most promising among the two candidate CDs, although some stability issues still need to be addressed. This result was somewhat expected, and is in agreement with experimental evidence.^43−46^ For this reason, further studies will be performed to investigate its properties against aggregation, both in the bulk and at interfaces, with special emphasis on the air–water system, where GCSF is known to easily undergo aggregation.^76,77^ An understanding of these basic mechanisms could be of considerable help, especially when investigating the stabilizing properties of excipients against aggregation.

In the present work, a model protein (GCSF) has been selected for the availability of experimental data and its small size that speeds up convergence of the simulations. However, we would expect the results obtained to be generally applicable and, therefore, to apply also to larger or more pharmaceutically relevant proteins. In the case of more surface active biologics, the HPβCD molecules may however be less effective in preventing adsorption to the air surface.

## Supporting Information Available

Convergence of simulations; β-parameter profiles (preferential exclusion from the protein hydration layer) and preferential orientation toward the protein; inclusion of peptide residues in CDs hydrophobic cavity; density profiles at interfaces and corresponding snapshots; free energy surfaces for α-helix content at the interfaces; and graphical representation of conformational changes (*Q*_res_) (PDF)

## ABBREVIATIONS

CD(s): cyclodextrin(s)
βCD: bCD, β-cyclodextrin
HPβCD: HPbCD, 2-hydroxypropyl-β-cyclodextrin
MD: molecular dynamics
MetaD: metadynamics
GCSF: granulocyte colony stimulating factor
αCD: α-cyclodextrin
γCD: γ-cyclodextrin
sim.: simulation
A/W: air–water
I/W: ice–water
PBMetaD: parallel bias metadynamics
CV(s): collective variable(s)
MW(s): multiple walker(s)
*R*_g_: radius of gyration
FES: free energy surfaces
(d)RMSD: (distance) root mean square deviation
IgG: immunoglobulin G
APR: aggregation prone region
