# Supplementary material for: The Role of Cyclodextrins against Interface-Induced Denaturation in Pharmaceutical Formulations: A Molecular Dynamics Approach
Source: Mol Pharm. 2021 May 17;18(6):2322–33. doi: 10.1021/acs.molpharmaceut.1c00135 (PMC8289300; doi:10.1021/acs.molpharmaceut.1c00135)
Supplement: Supplementary file 1 — mp1c00135_si_001.pdf [file mp1c00135_si_001.pdf]

Supporting Information for:

The Role of Cyclodextrins against Interface-Induced Denaturation in Pharmaceutical Formulations: A Molecular Dynamics Approach

Marcello Rospiccio[†], *Andrea Arsiccio*[†], *Gerhard Winter*[§], *Roberto Pisano* [*†]

[†]Molecular Engineering Laboratory, Department of Applied Science and Technology, Politecnico di Torino, Corso Duca degli Abruzzi 24, Torino 10129, Italy

[§]Department of Pharmacy, Ludwig-Maximilians-University, 81377 Munich, Germany

[*]Corresponding author: roberto.pisano@polito.it

Section S1. Convergence of simulations

In this section we show the convergence of both unbiased and biased simulations, with reference to the nomenclature of Table 1 (see main text). For unbiased simulations we evaluated the evolution over time of the number of CDs within 2 nm of distance from the GCSF surface (sim. 3a) and from the ice surface (sim. 9b). It can be observed that the number of molecules in said region of space converges after a few ns, and is completely equilibrated in the last part of the trajectory used for analysis (Figure S1). Therefore, we can assume that the systems under exam have reached an equilibrium state.

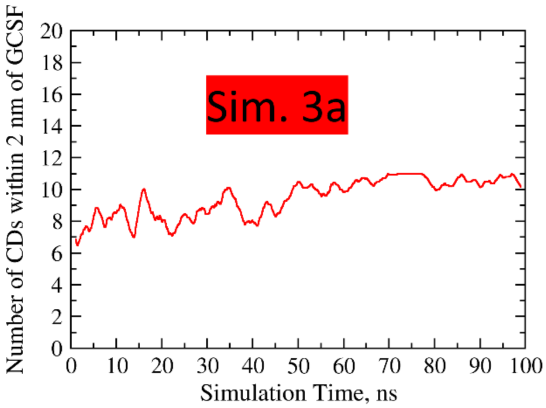

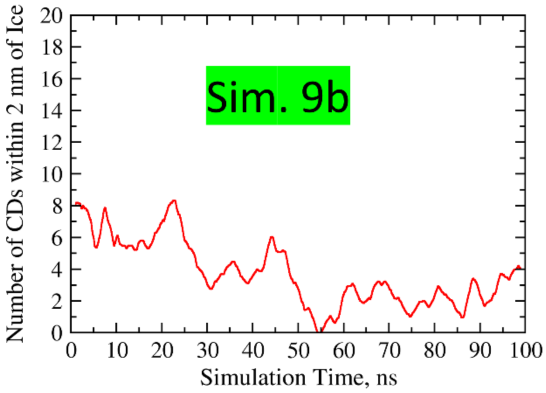

Figure S1. Convergence of unbiased simulations. Distribution of CDs within the box during sim. 3a and sim. 9b respectively. Color code: Red, aqueous bulk, 300 K. Green, Ice-water interface (I/W).

To prove the convergence of PBMetaD simulations (biased simulations, from 11 to 18 in Table 1), we show the variation of the collective variables (here we only report the results for the α-helix

content) over the last 10% of the simulation time. As seen in Figure S2, such variations are quite modest, therefore the simulations have converged.

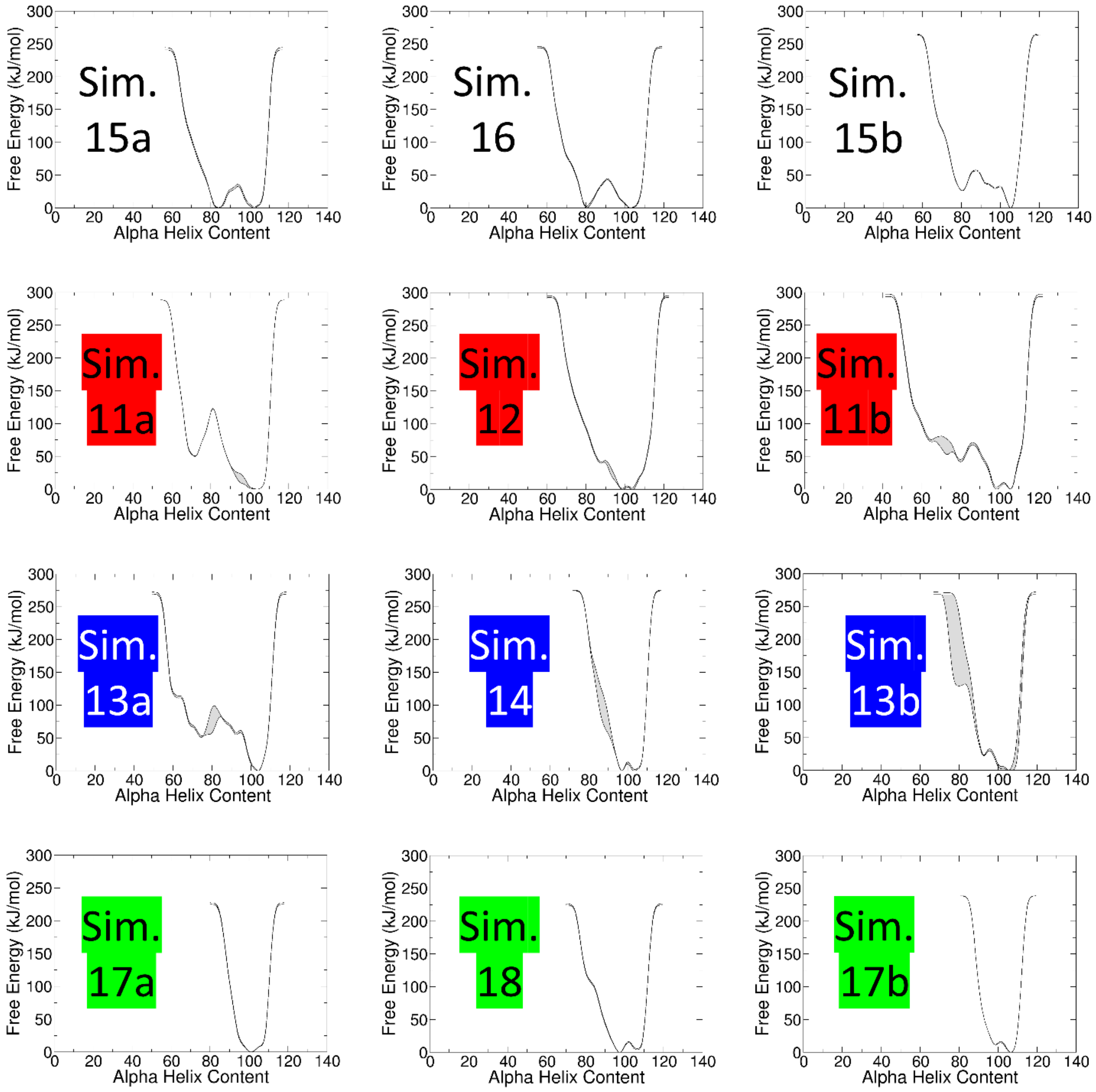

Figure S2. Convergence of biased simulations. Free energy content (kJ/mol) as a function of the α-helix content of GCSF. The numbers of each simulation refer to Table 1. Color code: White, A/W interface. Red, aqueous bulk, 300 K. Blue, aqueous bulk, 260 K. Green, I/W interface.

Section S2. CDs preferentially interact with the protein

The β-parameter profile,

$$\beta(r) = \frac{n_{EXC}(r)/n_W(r)}{n_{EXC}(\infty)/n_W(\infty)} \qquad \text{(S1)}$$

was evaluated in order to compare the interactions between excipients and the protein, as done already in previous works[1]. Here, $n_{EXC}(r)$ and $n_W(r)$ indicate the number of excipient and water molecules, respectively, within a distance r from the protein surface, while $n_{EXC}(\infty)$ and $n_W(\infty)$ are the total number of excipient/water molecules within the box. If the β-parameter is smaller than 1 at small values of r, the excipient is considered to be preferentially excluded from the hydration layer of the protein.

The behavior of GCSF in the bulk solution, at 300 and 260 K, was first investigated (sim. 3, 5 in Table 1). The β-parameter profiles (Figure S3a,b) suggested that βCD is preferentially included within the surface hydration layer of GCSF, compared to HPβCD. This behavior could be explained by the hydrophobic nature of GCSF, whose solvent accessible surface area is mostly non-polar (about 54% of GCSF surface in the native fold is non-polar). The hydrophobicity of βCDs cavities[2] (both the native and functionalized ones) could therefore be the driving force for the observed interaction with the protein. However, the analysis of the included peptide residues revealed that both excipients were able to host also polar uncharged and charged moieties (see the following section S2, and Figures S3, S4). The CDs seem therefore to display a general affinity towards the backbone group, which would still allow interactions with specific sidechains, especially the aromatic ones[3].

Interestingly, simulations at 260 K indicated a lower tendency to preferential inclusion for both CDs, as well as fewer residues inclusions. In particular, non-polar amino acids were less included, and this could be explained considering the hydrophobic effect[4], that suggests increased water-hydrophobes interaction at low temperature. Moreover, the CDs tended to interact more with each other at low temperatures, forming aggregates, and this may also account for the observed reduction in the ability of these excipients to interact with the protein.

The interactions between CDs and GCSF were also evaluated at the air and ice-water interfaces (sim. 7, 9). At the air surface, both excipients showed once again preferential inclusion, although the βCD molecules were less included with respect to the bulk case (Figure S3c). Accordingly, the native CD included fewer peptide residues, while the functionalized form exhibited more inclusions (Figure S7).

At the ice surface, the β-parameter profiles suggested that the excipients were still preferentially included, although in this case βCD was less included than HPβCD (Figure S3d). Similarly, peptide residues were less included by the native CD (Figure S8), if compared to the bulk system at 260 K (Figure S6). A more in-depth analysis was performed, in order to assess the existence of any preferential orientations. We evaluated the radial distribution functions for the rims of both CDs and observed that in almost all cases the secondary rim was the closest to the protein surface (Figure S4). The only exception to this tendency was observed for the βCD formulation at the ice-water interface, where the primary rim was found to be closer. This intriguing change in the preferred orientation may explain the inversion in the preferential inclusion trend, with the native CD being less included than the functionalized one (Figure S3d). However, the nature of such behavior is still unclear, and warrants further investigation.

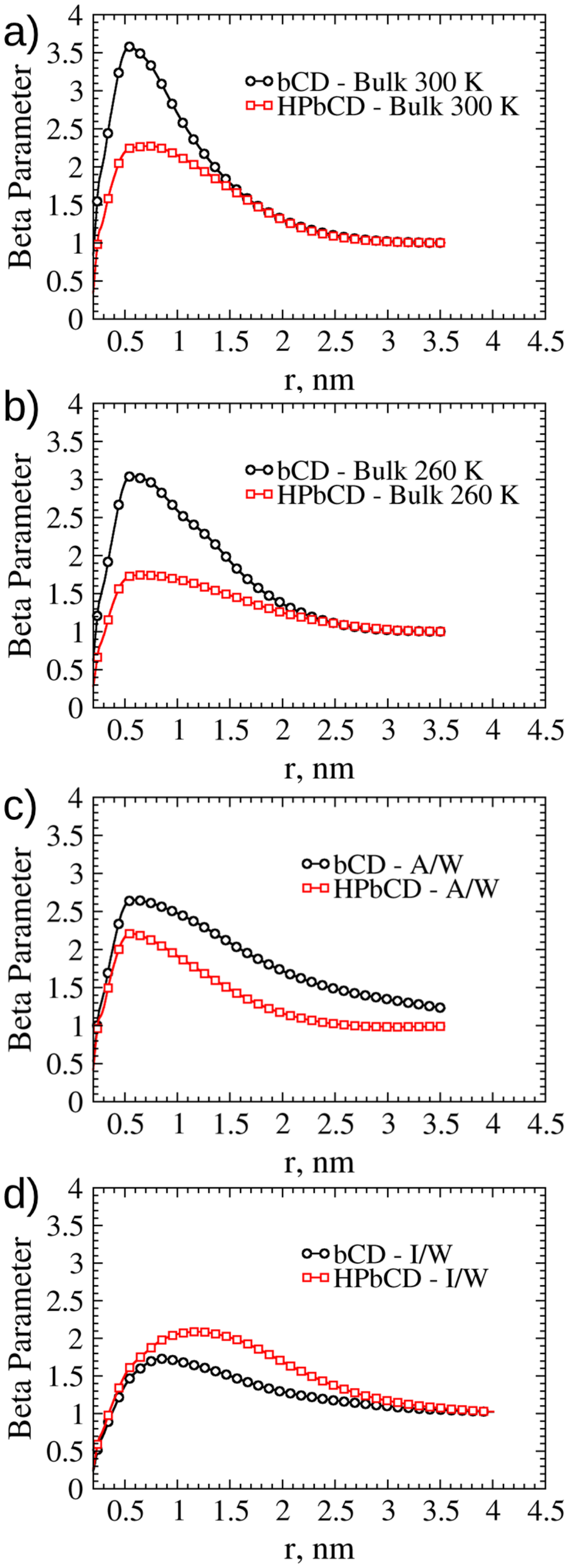

Figure S3. β-parameter profiles for both cyclodextrins. a) Bulk systems at 300 K (sim. 3). b) Bulk systems at 260 K (sim. 5). c) Systems at the air-water interface (A/W, sim. 7). d) Systems at the ice-water interface (I/W, sim. 9).

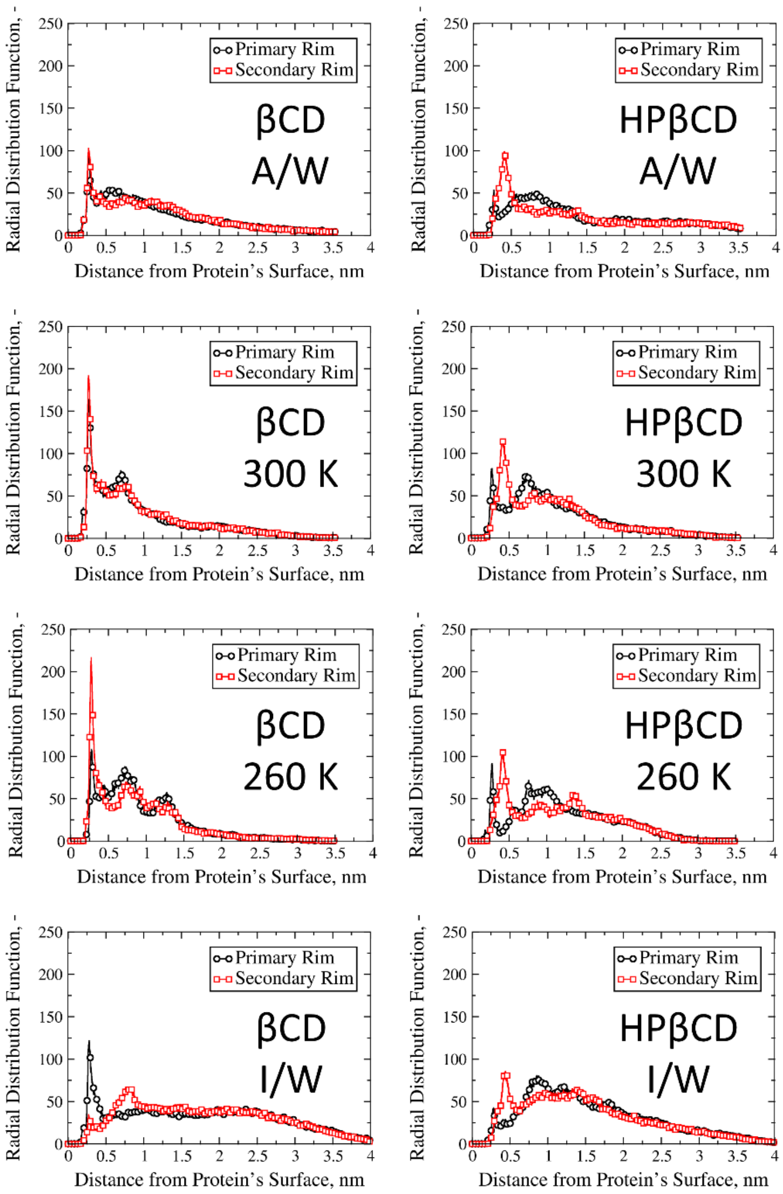

Figure S4. Radial distribution functions for the rims of all CD systems, unbiased simulations (simulations 3, 5, 7, 9 from Table 1).

Section S3. Inclusion of peptide residues in CDs hydrophobic cavity

Residues inclusions were evaluated by counting the number of frames in which the atoms of each residue were in close proximity of the cyclodextrins (CDs) cavities. More specifically, a spherical neighborhood having 0.5 nm radius, and centered in the center of mass of the cyclodextrins, was used to define the region where inclusion occurs. The number of frames during which inclusion was

observed was then normalized to allow a more direct comparison of the different simulations, dividing by the total number of analyzed frames and the number of cyclodextrins in the system. The results obtained are shown in Figures S3-S6.

This analysis allowed us to evaluate which type of CD interacted the most with GCSF in a given system, and also enabled us to identify the amino acids with the highest level of inclusion.

In general, we observed a rather interesting and unexpected result: CDs included not only moieties of aromatic sidechains (a well-known observation in the literature), but also other types of residues. Such behavior could be explained by a general inclusion mechanism based on the interaction between the cavities and the backbone of the residues. In fact, the carbon chain, common to all amino acids, may have a modest affinity towards the hydrophobic environment of CDs cavities. However, whenever this was energetically favorable, interaction could switch from the backbone to the sidechain. Also, it is necessary to consider the effects of the protein structure: even if some species of residues were more likely to interact, they were buried within the core of the molecule and were thus not solvent accessible. This is particularly true for hydrophobic amino acids, which at room temperature are more likely to be found in the internal core of a protein. Finally, steric hindrance and unfavorable conformations may also limit the possibility of inclusions.

Further studies on the interactions between amino acids and CDs are necessary to elucidate the underlying mechanisms, and this will be the subject of future investigations.

βCD – GCSF – Bulk 300 K

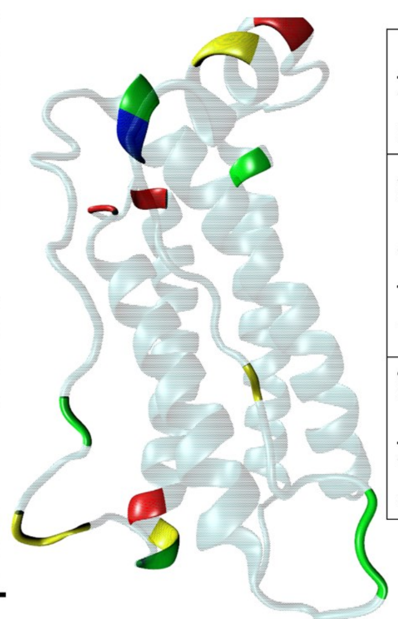

Residue N°	Inclusion, %	Residue
129	4.051%	ALA
130	4.822%	LEU
133	0.756%	THR
145	0.834%	GLN
146	4.893%	ARG
46	2.373%	GLU
36	0.375%	CYS
50	2.489%	LEU
61	0.401%	LEU
66	0.567%	SER
67	1.044%	GLN
93	3.540%	GLU
98	0.379%	GLU
122	1.907%	GLU
124	0.389%	LEU
125	4.479%	GLY

POSITIVELY CHARGED
NEGATIVELY CHARGED
POLAR
NON-POLAR

HPβCD – GCSF – Bulk 300 K

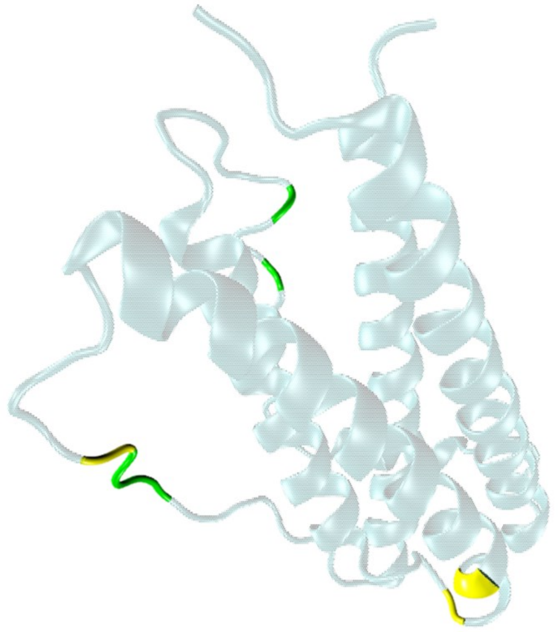

Residue N°	Inclusion, %	Residue
132	0.618%	PRO
133	1.125%	THR
134	2.860%	GLN
135	2.075%	GLY
62	0.812%	SER
65	0.421%	SER
97	1.283%	PRO
101	0.917%	PRO

Figure S5. Included residues, type of residue, inclusion probability (percentage), and snapshots of included residues for the GCSF formulations at 300 K in the bulk.

βCD – GCSF – Bulk 260 K

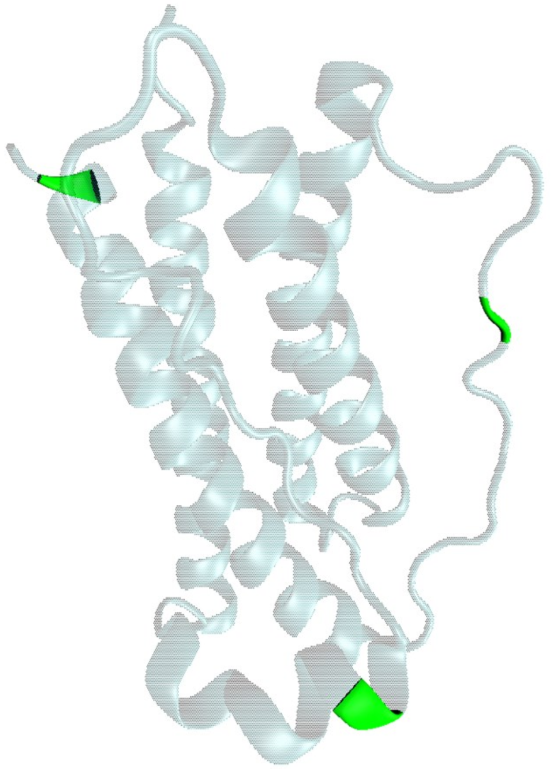

Residue N°	Inclusion, %	Residue
133	8.074%	THR
173	9.091%	GLN
53	1.117%	SER

HPβCD – GCSF – Bulk 260 K

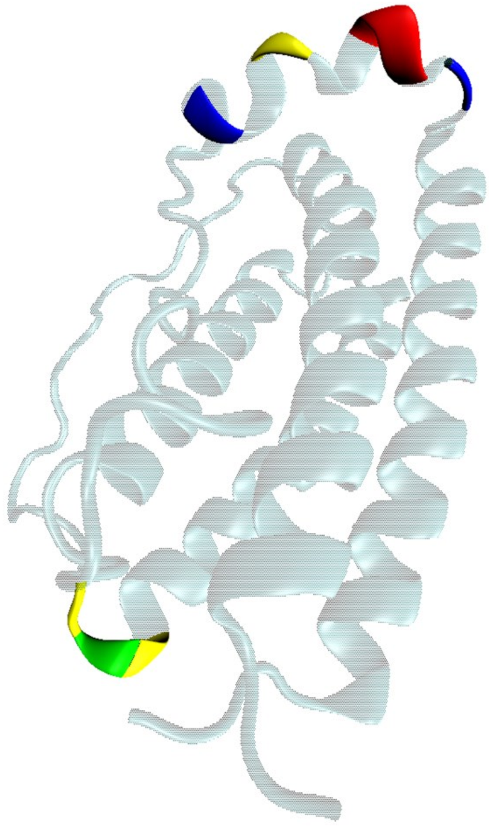

Residue N°	Inclusion, %	Residue
43	0.073%	HIS
45	0.379%	GLU
46	0.136%	GLU
49	0.299%	LEU
52	0.248%	HIS
124	0.283%	LEU
125	0.748%	GLY
126	1.128%	MET

POSITIVELY CHARGED
NEGATIVELY CHARGED
POLAR
NON-POLAR

Figure S6. Included residues, type of residues, inclusion probability (percentage) and snapshots of included residues for the GCSF formulations at 260 K in the bulk.

βCD – GCSF – Air-Water

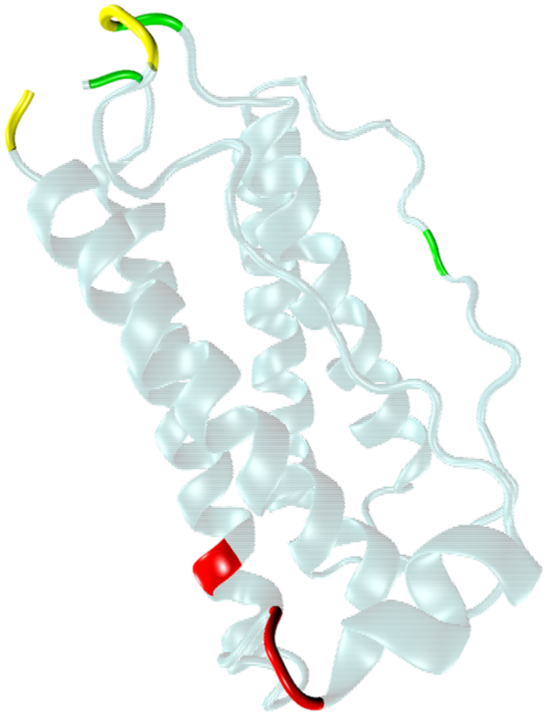

Residue N°	Inclusion, %	Residue
133	0.551%	THR
8	0.173%	SER
174	0.228%	PRO
33	1.939%	GLU
45	0.457%	GLU
46	0.329%	GLU
68	1.203%	ALA
69	6.073%	LEU
70	0.946%	GLN

POSITIVELY CHARGED
NEGATIVELY CHARGED
POLAR
NON-POLAR

HPβCD – GCSF – Air-Water

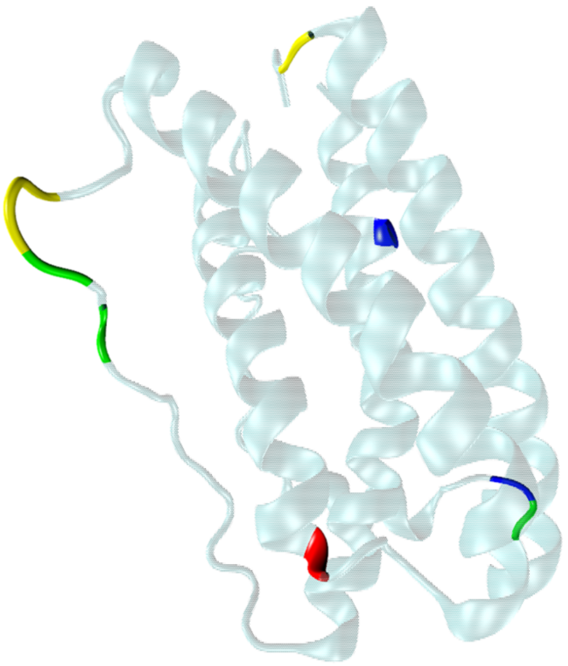

Residue N°	Inclusion, %	Residue
129	3.673%	ALA
130	6.859%	LEU
131	3.463%	GLN
133	0.239%	THR
10	0.396%	PRO
166	0.384%	ARG
42	2.840%	CYS
43	0.574%	HIS
93	0.501%	GLU

Figure S7. Included residues, type of residues, inclusion probability (percentage) and snapshots of included residues for the GCSF formulations at the air-water interface.

βCD – GCSF – Ice-Water

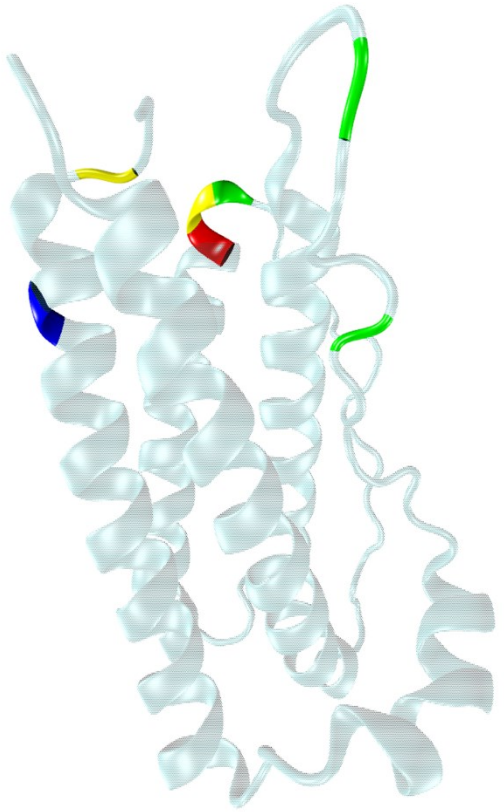

Residue N°	Inclusion, %	Residue
67	0.845%	GLN
10	1.735%	PRO
16	0.413%	LYS
124	1.538%	LEU
123	2.476%	GLU
125	0.665%	GLY
62	0.136%	SER

POSITIVELY CHARGED
NEGATIVELY CHARGED
POLAR
NON-POLAR

HPβCD – GCSF – Ice-Water

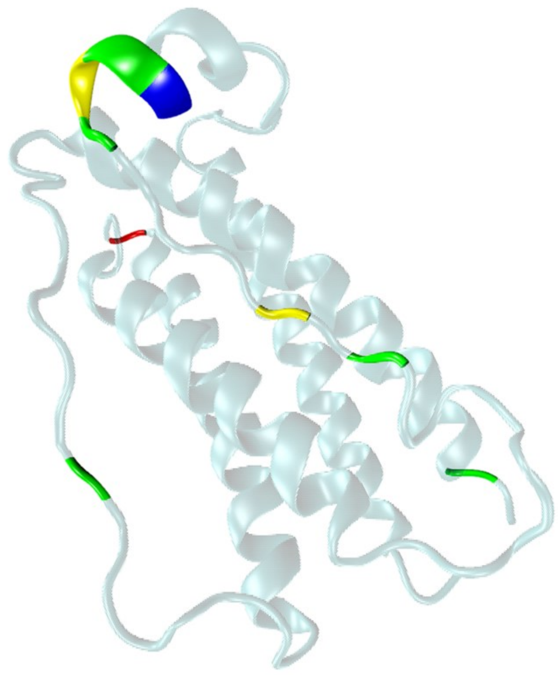

Residue N°	Inclusion, %	Residue
98	1.120%	GLU
133	0.287%	THR
173	0.082%	GLN
52	0.280%	HIS
62	0.218%	SER
54	0.208%	LEU
55	2.444%	GLY
60	0.343%	PRO
53	0.241%	SER

Figure S8. Included residues, type of residues, inclusion probability (percentage), and snapshots of included residues for the GCSF formulations at the ice-water interface.

Section S4. Interfaces – MD simulations

To study the behavior of CDs in proximity of an interface, we computed the normalized density profiles for both cyclodextrins and GCSF, expressing them as a function of the distance from the interface. Initially, we evaluated the profiles for the interfacial systems without proteins (sim. 1, 2 in Table 1). The behavior at the air-water interface was different for the two CDs. βCD accumulated in bulk (Figure S9a), with no preferential orientation of the rims (Figure S9a), as also shown in Figure S9c. Instead, HPβCD distinctly accumulated at the air-water interface (Figure S9b), with its hydrophobic cavity (secondary rim) towards the gaseous phase (Figure S9b), as shown in Figure S9d. The same behavior was observed in the systems with GCSF at the air-water interface, as displayed in Figure S10. The corresponding density profiles are shown in the main text (Figure 2).

The same procedure was used for the ice-water interface. The behavior of the CDs was the same both in presence and absence of the GCSF, i.e. accumulation in the aqueous bulk was observed, with no preferential orientation of the rims. The density profiles of the systems with and without protein are shown in Figure 3 of the main text and Figure S11, respectively.

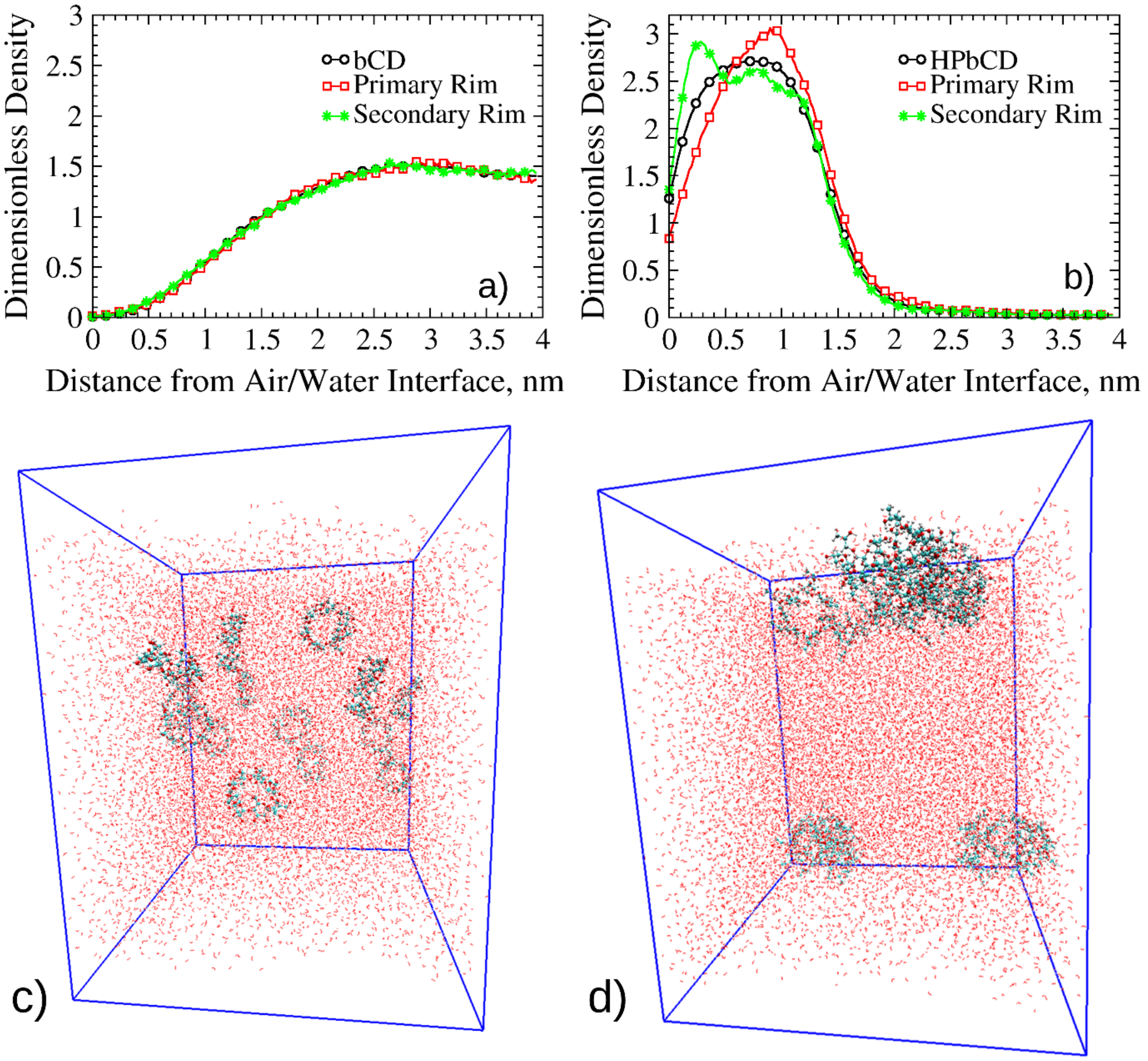

Figure S9. Cyclodextrins systems at the air-water interface. a) Density profiles for βCD and its rims. b) Density profiles for HPβCD and its rims. c) Snapshot of the βCD/air-water system. The blue lines indicate the limits of the simulation box. d) Snapshot of the HPβCD/air-water system. The blue lines indicate the limits of the simulation box. The snapshots were realized with Visual Molecular Dynamics (VMD)[5].

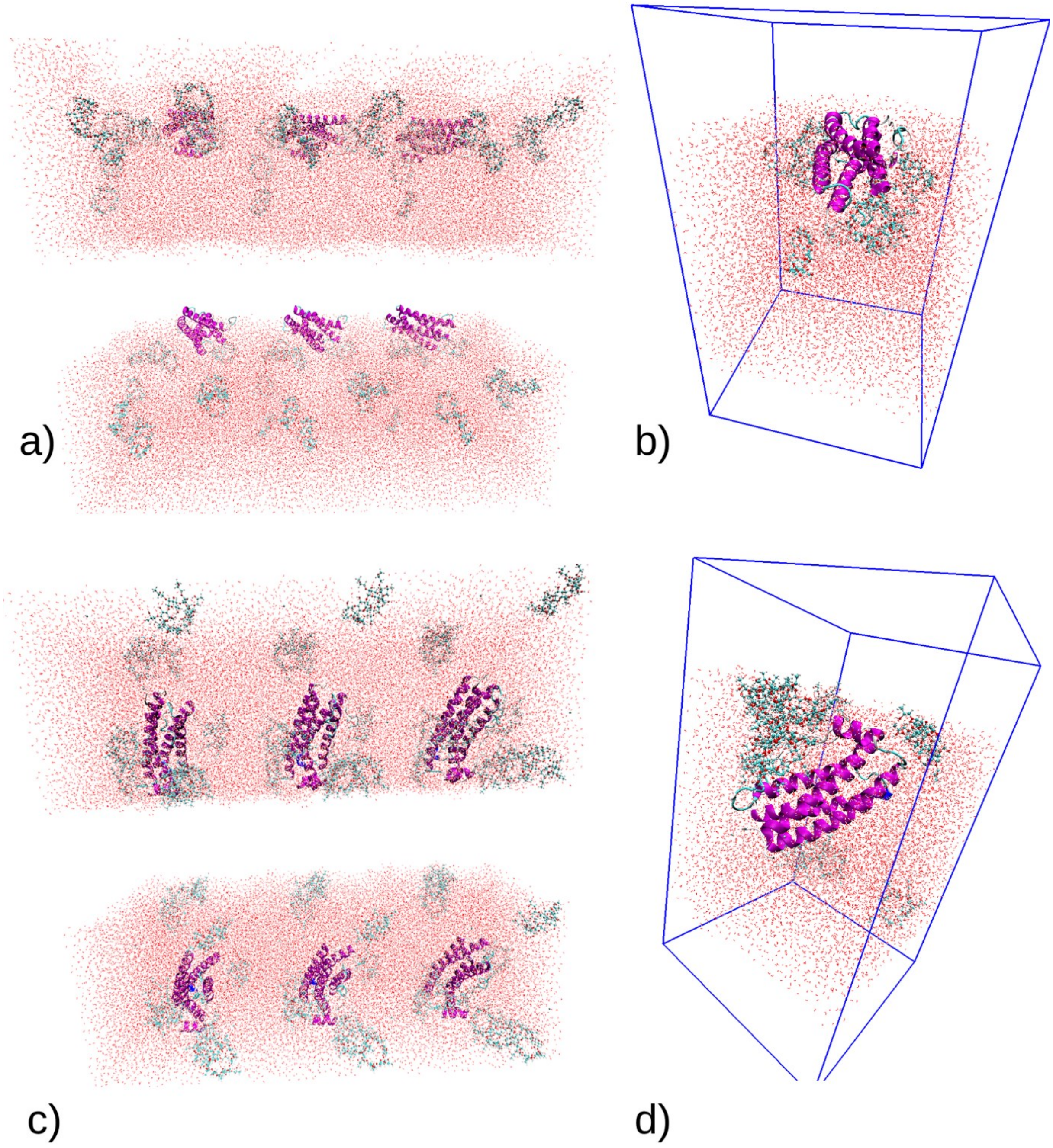

Figure S10. a) Extended periodic images of the βCD-GCSF/air-water system. b) Single-box representation of the βCD-GCSF/air-water system. The blue lines indicate the limits of the system. c) Extended periodic images of the HPβCD-GCSF/air-water system. b) Single-box representation of the HPβCD-GCSF/air-water system. The blue lines indicate the limits of the system. The snapshots were realized with Visual Molecular Dynamics (VMD)[5].

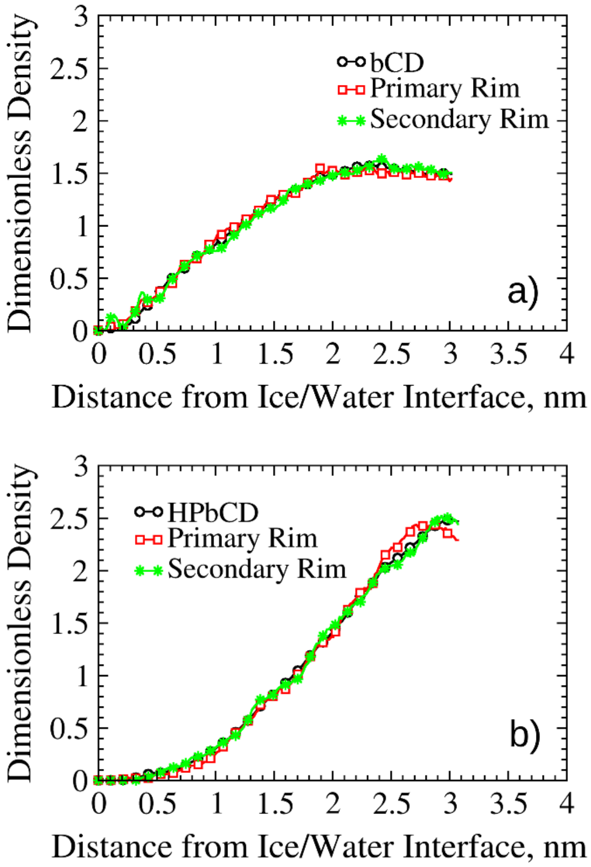

Figure S11. Cyclodextrins systems at the ice-water interface. a) Density profiles for βCD and its rims. b) Density profiles for HPβCD and its rims.

Section S5. α-helix content at the interfaces: Free Energy Surfaces

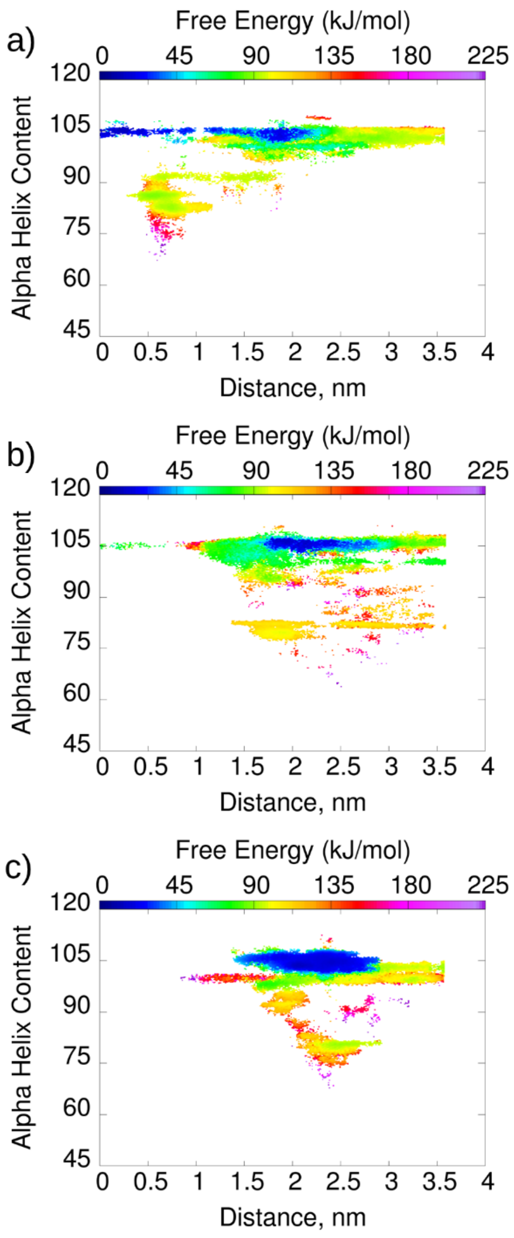

Figure S12. Free energy surfaces for the GCSF systems at the air-water interface (simulations 15 and 16 in Table 1). The energy landscape of the system is projected as a function of the α-helix content of the protein and the distance from the interface. a) βCD-GCSF formulation. b) GCSF without excipients. c) HPβCD-GCSF formulation.

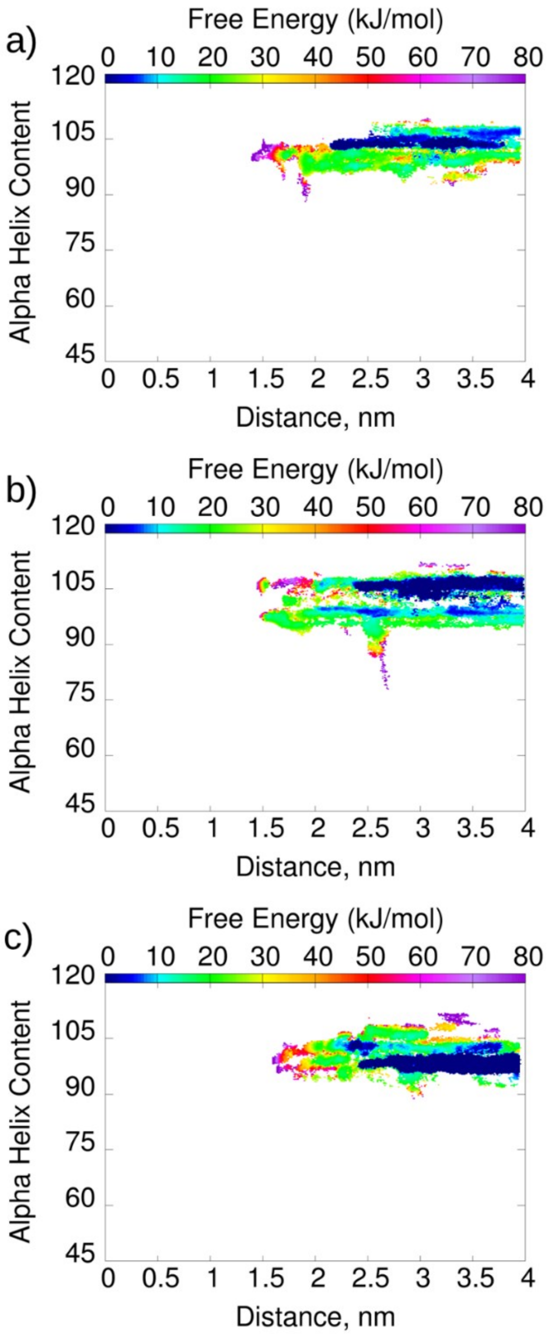

Figure S13. Free energy surfaces for the GCSF systems at the ice-water interface (simulations 17 and 18 in Table 1). The energy landscape of the system is projected as a function of the α-helix content of the protein and the distance from the interface. a) βCD-GCSF formulation. b) GCSF without excipients. c) HPβCD-GCSF formulation. The scale of the energy bar is different than for the air-water systems (Figure S12), to better visualize energetic differences.

Section S6. Conformational changes: Q_{res} graphical representation

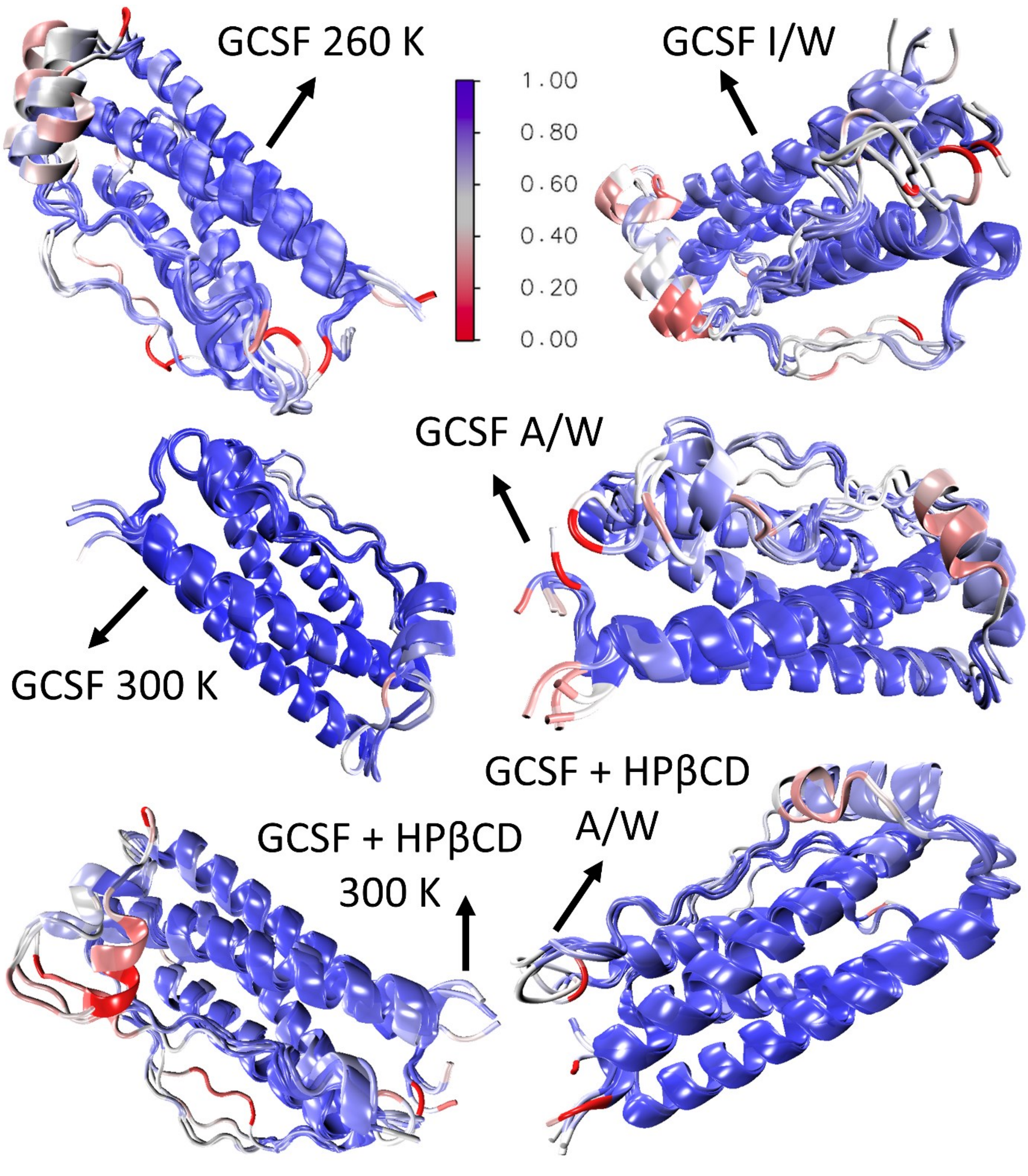

Figure S14. Graphical representation of Q_{res} values on aligned protein structures. A/W: air-water interface; I/W: ice-water interface; 300 K: aqueous bulk at 300 K; 260 K: aqueous bulk at 260 K. Q_{res} values progressively decrease from 1 to 0 according to the color bar.

AUTHOR INFORMATION

Corresponding Author

*Email: roberto.pisano@polito.it

Notes

The authors declare no competing financial interest.

ACKNOWLEDGMENTS

Computational resources were provided by HPC@POLITO, a project of Academic Computing within the Department of Control and Computer Engineering at the Politecnico di Torino (http://hpc.polito.it), and by CINECA under the ISCRA initiative (DisCyc-HP10CQROS1, MDCDprot-HP10C4MJK3 and CD-AA-FF-HP10C1LPRF).

ABBREVIATIONS

CD(s), cyclodextrin(s); βCD, bCD, β-cyclodextrin; HPβCD, HPbCD, 2-hydroxypropyl-β-cyclodextrin; MD, Molecular Dynamics; MetaD, Metadynamics; GCSF, Granulocyte colony stimulating factor; αCD, α-cyclodextrin; γCD, γ-cyclodextrin; sim., simulation; A/W, air-water; I/W, ice-water; PBMetaD, Parallel Bias Metadynamics; CV(s), collective variable(s); MW(s) multiple walker(s); R_g, radius of gyration; FES, free energy surfaces; sasa, solvent accessible surface area; RMSD, root mean square deviation;

REFERENCES

(1) Arsiccio, A.; Paladini, A.; Pattarino, F.; Pisano, R. Designing the Optimal Formulation for Biopharmaceuticals: A New Approach Combining Molecular Dynamics and Experiments. *J. Pharm. Sci.* **2019**, *108* (1), 431–438. https://doi.org/10.1016/j.xphs.2018.09.002.

(2) Khuntawee, W.; Karttunen, M.; Wong-Ekkabut, J. A Molecular Dynamics Study of

Conformations of Beta-Cyclodextrin and Its Eight Derivatives in Four Different Solvents. *Phys. Chem. Chem. Phys.* **2017**, *19* (35), 24219–24229. https://doi.org/10.1039/c7cp04009a.

(3) Horský, J.; Pitha, J. Inclusion Complexes of Proteins: Interaction of Cyclodextrins with Peptides Containing Aromatic Amino Acids Studied by Competitive Spectrophotometry. *J. Incl. Phenom. Mol. Recognit. Chem.* **1994**, *18* (3), 291–300. https://doi.org/10.1007/BF00708735.

(4) Dias, C. L.; Ala-Nissila, T.; Wong-ekkabut, J.; Vattulainen, I.; Grant, M.; Karttunen, M. The Hydrophobic Effect and Its Role in Cold Denaturation. *Cryobiology* **2010**, *60* (1), 91–99. https://doi.org/10.1016/j.cryobiol.2009.07.005.

(5) Humphrey, W.; Dalke, A.; Schulten, K. VMD: Visual Molecular Dynamics. *J. Mol. Graph.* **1996**, *14* (1), 33–38. https://doi.org/10.1016/0263-7855(96)00018-5.
